# *Drosophila* imaginal disc growth factor 2 is a trophic factor involved in energy balance, detoxification, and innate immunity

**DOI:** 10.1038/srep43273

**Published:** 2017-02-23

**Authors:** Vaclav Broz, Lucie Kucerova, Lenka Rouhova, Jana Fleischmannova, Hynek Strnad, Peter J. Bryant, Michal Zurovec

**Affiliations:** 1 Institute of Entomology, Biology Centre CAS, Branisovska 31, 370 05 Ceske Budejovice, Czech Republic; 2 Faculty of Science, University of South Bohemia, Branisovska 31, 370 05 Ceske Budejovice, Czech Republic; 3 Institute of Molecular Genetics CAS, Videnska 1083, 142 20 Prague 4, Czech Republic; 4 Developmental & Cell Biology, School of Biological Sciences, University of California, Irvine, USA

## Abstract

*Drosophila* imaginal disc growth factor 2 (IDGF2) is a member of chitinase-like protein family (CLPs) able to induce the proliferation of imaginal disc cells *in vitro*. In this study we characterized physiological concentrations and expression of IDGF2 *in vivo* as well as its impact on the viability and transcriptional profile of *Drosophila* cells *in vitro*. We show that IDGF2 is independent of insulin and protects cells from death caused by serum deprivation, toxicity of xenobiotics or high concentrations of extracellular adenosine (Ado) and deoxyadenosine (dAdo). Transcriptional profiling suggested that such cytoprotection is connected with the induction of genes involved in energy metabolism, detoxification and innate immunity. We also show that IDGF2 is an abundant haemolymph component, which is further induced by injury in larval stages. The highest IDGF2 accumulation was found at garland and pericardial nephrocytes supporting its role in organismal defence and detoxification. Our findings provide evidence that IDGF2 is an important trophic factor promoting cellular and organismal survival.

The cells of higher eukaryotes integrate mitogenic, differentiating, stress and defence signals with aging or nutrient status to regulate growth and proliferation. Several growth factors have been shown to be involved in the growth of *Drosophila* imaginal disc cells, including epidermal growth factor (EGF), proteins of the insulin family, adenosine deaminase growth factors (ADGFs)^1^,^2^ and imaginal disc growth factors (IDGFs)^3^. It is important to fill the current gaps in our knowledge of IDGFs, which are unusual among the growth factors since they are present in insect haemolymph in large concentrations and seem to integrate signals needed for growth, cell viability, and the innate immune response.

IDGFs comprise a small family of six secreted glycoproteins with a molecular weight of about 47 kDa and are identified as active growth factors from the conditioned media of the *Drosophila* cell lines^4^,^5^. They belong to the chitinase-like proteins (CLPs), showing a mutual sequence identity of approximately 50%, and are produced by the larval fat body and haemocytes^5^,^6^. IDGFs are structurally related to a large family of 18 glycosyl hydrolases known in both vertebrates and invertebrates (15–25% amino acid sequence identity), which include chitinases and chitinase-like proteins^7^. Unlike chitinases, IDGFs are not active enzymes since they carry an amino acid substitution that is known to abrogate chitinase catalytic activity, but they retain the ability to bind carbohydrates^7^,^8^,^9^. CLPs have been reported to regulate responses to bacteria, cell growth, inflammation, and remodelling in various organisms by a mechanism that is still poorly understood^10^,^11^,^12^.

IDGF2 is the best characterized *Drosophila* IDGF, having its crystal structure determined^7^. High levels of *Idgf2* mRNA expression have been reported in the yolk cytoplasm of the early embryo. High levels of IDGF2 protein have been detected in the haemolymph of *Drosophila* third instar larvae^13^,^14^ as well as in the larval fat body and salivary glands^5^. In adults, *Idgf2* mRNA has been detected in nurse cells and oocytes^5^. *Idgf2* was also identified as one of the genes upregulated in the early stages of imaginal disc regeneration^15^.

Recombinant IDGF2 showed a dose-dependent effect on the growth of imaginal disc Cl.8+ cells in supplement-free media (SFM) when used together with bovine insulin^5^. Vertebrate insulin has earlier been shown to activate the *Drosophila* insulin receptor^16^, and, based on the proposed cooperation between IDGF2 and insulin in stimulating imaginal disc cell growth, it has been suggested that IDGF2 might function as a cofactor of *Drosophila* insulin^5^.

Here, we examined the effects of IDGF2 on cell growth in tissue culture cells *in vitro* and searched for the mechanisms involved. We show that recombinant IDGF2 at levels corresponding to its haemolymph concentration supports the survival of Cl.8+ cells independently of insulin. The effects of recombinant IDGF2 include protection against cell death caused by serum deprivation, as well as against elevated levels of Ado, dAdo and some xenobiotics in serum-free conditions. We found that the highest accumulation of IDGF2 protein *in vivo* was in pericardial and garland nephrocytes that contribute to detoxification of the insect haemolymph. Furthermore, IDGF2 is induced by injury *in vivo* and activates the expression of a number of target genes involved in the energy metabolism, detoxification, and the innate immune response.

## Results

### Recombinant IDGF2 promotes the growth of Cl.8+ cells *in vitro*

**Figure 1:**
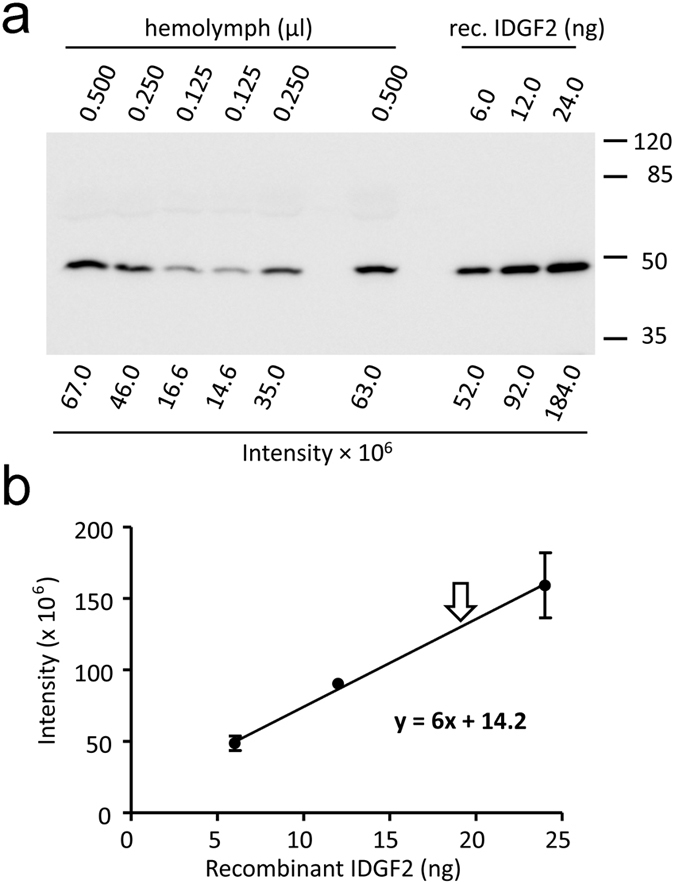
Quantification of IDGF2 protein in *Drosophila* haemolymph. (**a**) Representative western blot image showing 6–24 ng purified recombinant IDGF2 together with haemolymph samples separated on a 10% PAGE. IDGF2 was detected by anti-IDGF2 antibody. (**b**) Calibration curve derived by plotting the band density of haemolymph IDGF2 against known amounts of recombinant IDGF2 protein (based on three experiments). For haemolymph isolation, wandering L3 larvae were collected, larvae were surface sterilized in 70% ethanol and excess fluid was blotted off on paper towel. 10 larvae were used for isolation. The larvae were opened by gently pulling the epidermis apart with forceps. The haemolymph was collected with a fine glass pipette and immediately frozen. The haemocytes were not removed.

In order to examine the effect of the growth factor IDGF2 on the proliferation of *Drosophila* Cl.8+ cells, we prepared a recombinant IDGF2 protein in a baculovirus expression system. First, we used the recombinant IDGF2 to determine the concentration of native IDGF2 in *Drosophila* haemolymph *in vivo* (Fig. 1). The results of three independent experiments indicated that the concentration of IDGF2 in the haemolymph is 19 ± 3 ng/μl (approximately 400 nM).

**Figure 2:**
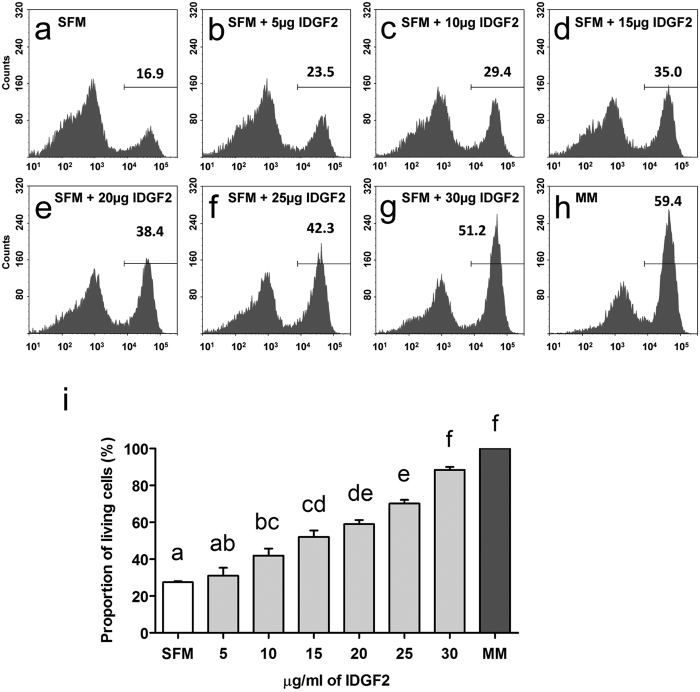
IDGF2 effect on the mitochondrial membrane potential (ΔΨm) of Cl.8+ cells incubated 36 hrs in SFM determined by TMRE staining and flow cytometry. Higher than physiological doses of IDGF2 (more than 20 μg/ml) are needed for Cl.8+ cell survival during a longer incubation period. (**a**) Control Cl.8+ cells in supplement free media (SFM); (**b–g**) Cl.8+ cells in increasing concentrations of recombinant IDGF2 (5, 10, 15, 20, 25 and 30 μg/ml, respectively). (**h**) Cl.8+ cells in chemically defined medium (MM). Living cells with active mitochondria sequester the TMRE stain (right peaks), while TMRE-negative cells undergo apoptosis (left peaks). Increasing concentrations of IDGF2 led to a higher proportion of living cells. The total cell population was gated on TMRE-positive cells, and the numbers represent the proportion of these cells. (**i**) Dose dependent effect of IDGF2 on Cl.8+ cell viability in SFM shown as the proportion of control cells in MM. The graph summarizes the three cell cytometry experiments and the data is presented as mean ± SEM. Significant differences were evaluated by ANOVA followed by Tukey test and are indicated by different letters (p < 0.05).

Kawamura *et al*.^5^ showed that the addition of recombinant IDGF2 promotes the survival and growth of Cl.8+ cells in a supplement-free medium (without FBS or fly extract), but containing yeast extract (SFM). To verify the function of recombinant IDGF2, we treated the Cl.8+ cells in SFM with physiologically-relevant IDGF2 concentrations and assessed their viability by monitoring changes in mitochondrial membrane potential (ΔΨm) and morphology. As shown in Fig. 2a, the TMRE readouts of cell treated for 36 hrs with SFM were characterized by a small proportion (16.9%) of living cells (right peak in the plot, Fig. 2a). Consistently, increasing concentrations of IDGF2 dose dependently increased cell viability (Fig. 2a–h and Fig. S1a–g). Our results show that higher than physiological concentrations of IDGF2 are needed for the survival of the Cl.8+ cells for longer incubation in SFM or chemically defined minimal medium (MM) (Fig. 2 and S1). These results correlated with the cell morphology, such that the cell population with reduced TMRE staining also had a lower FSC signal corresponding to a smaller cell size and an increased SSC signal indicating higher density characteristic for apoptotic cells (Fig. S1a’–g’).

Since the SFM contained an undefined component, yeast extract, we also prepared fully defined cell culture media (minimal media, MM) containing bovine insulin, but without yeast extract. Interestingly, as shown in Fig. S1, the Cl.8+ cells in MM survived better than in SFM showing a modest IDGF2 effect on both the ΔΨm (Fig. S1h–n) and cell morphology (Fig. S1h’–n’). Our results show that IDGF2 has dose-dependent protective effects in both types of growth media, which prevent loss of ΔΨm and morphological alterations.

**Figure 3:**
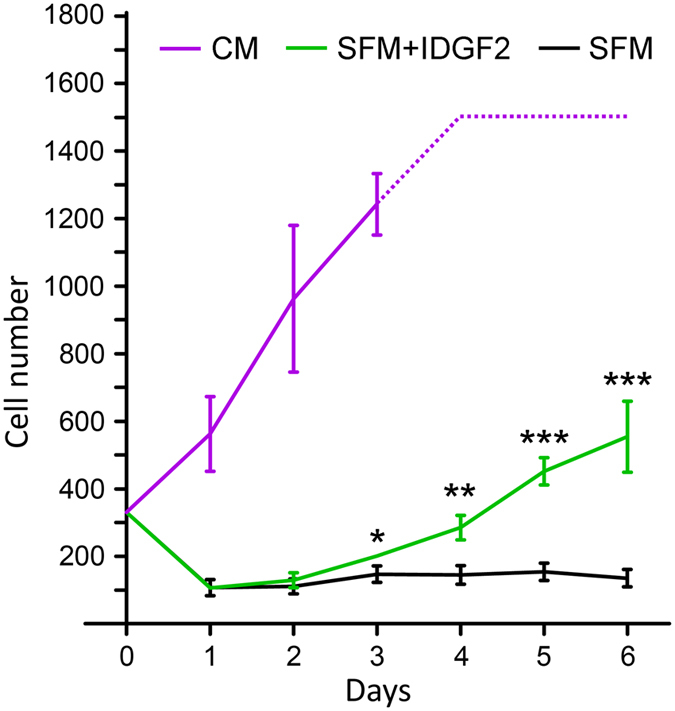
Effect of IDGF2 on the proliferation of *Drosophila* imaginal disc cells. The Cl.8+ were grown in different cell culture conditions. CM, complete medium; SFM - supplement-free medium (=medium containing yeast extract and insulin); SFM + IDGF2 - cells in SFM + IDGF2 (16 μg/ml). The growth/survival rates of cells were measured by the direct counting of cells using digital photographs of identical areas (0.8 × 0.8 mm) taken every 24 hrs. Each point represents the mean ± SEM (n = 3). Significant differences (*p < 0.05, **p < 0.01, ***p < 0.001) between SFM and SFM + IDGF2 treatments are indicated by asterisks and were evaluated by Student’s t-test.

We also compared the proliferation of Cl.8+ cells in SFM and SFM plus IDGF2 with the proliferation of these cells under standard conditions in complete media (CM, containing FBS, fly extract, yeast extract and insulin)^17^. We tested the effect of recombinant IDGF2 on cell division by direct cell counting (Fig. 3). The results show that Cl.8+ cells in SFM without IDGF2 did not grow and slowly died, while IDGF2 treatment (16 μg/ml) of such cells stimulated proliferation after a short lag phase in which about two thirds of the cells died but the rest grew with a doubling time of approximately 3 days. In contrast, the cells in the CM proliferated faster with a doubling time of approximately 2 days, most probably due to the nutrient-rich conditions.

Our results showed that the effect of recombinant IDGF2 on the survival and proliferation of Cl.8+ cells in SFM or MM was dose-dependent with concentrations of 10–30 μg/ml. The Cl.8+ cells in SFM required higher than physiological IDGF2 concentrations to show similar viability as the cells in MM. Interestingly, the addition of IDGF2 to Cl.8+ cells in CM caused only a marginal increase in ΔΨm (Fig. S2).

### IDGF2 exerts cytoprotective effects on Cl.8+ cells independent of insulin

**Figure 4:**
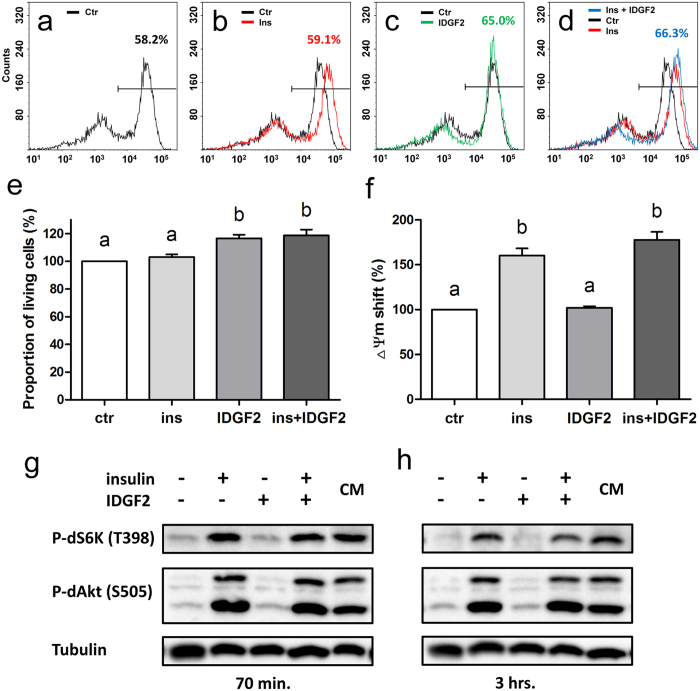
Effects of insulin and IDGF2 on Cl.8+ cells in chemically defined media. (**a–d**) Representative examples of flow cytometry plots of TMRE fluorescence of Cl.8+ cells incubated for 24 h in (**a**) MM (**b**) MM with insulin, (**c**) MM with IDGF2 and (**d**) MM with insulin and IDGF2. Numbers represent the proportion of viable cells. (**e**) The effects of insulin and IDGF2 on the proportion of living cells. Values represent the average percentage change in TMRE staining of living cells (right peaks) compared to control. The graph summarizes the three cell cytometry experiments shown in Fig. S3. (**f**) Effects of insulin and IDGF2 on the position of the right peak median. Values represent the average median increase in TMRE staining of living cells (right peaks) compared to control. The graph summarizes the three flow cytometry experiments shown in Fig. S3. Data in the graphs are presented as mean ± SEM. Significant differences were evaluated by ANOVA followed by Tukey test and are indicated by different letters (p < 0.05). (**g,h**) Western blot analysis comparing the levels of phosphorylated dS6K (T398) and dAkt/PKB (S505) in Cl.8+ cells grown in MM for 24 hours, followed by insulin (0.125 IU/ml) or IDGF2 (16 μg/ml) treatment. CM – cells grown in complete media. The cells were harvested after 70 min (**g**) and 3 hrs (**h**) incubation. The phosphorylation of dS6K and dAkt/PKB was significantly increased in insulin-treated cells, but not in IDGF2-treated cells. Tubulin detected with anti-alpha *tubulin* antibody was used as a loading control. Full-length blots are shown in Fig. S11.

Earlier report suggested that IDGF2 is a cofactor of *Drosophila* insulin-like peptides^5^. We therefore compared the effects of insulin and IDGF2 on the viability of Cl.8+ cells in insulin-free minimal medium (MM) using TMRE staining. The results show that Cl.8+ cells treated with IDGF2 had a significantly higher proportion of living cells (Fig. 4) than the control. In an example shown in Fig. 4c, the IDGF2 increased cell viability by about 8% (from 58.2% to 65.0%) while insulin showed almost no effect by itself as well as no synergy with IDGF2. Interestingly, while insulin treatment did not affect the proportion of living and dying cells, it significantly shifted the position of the right peak further right (increased the ΔΨm) (Fig. 4b,f). These results suggest that the responses to both growth factors are distinct and probably mediated by separate mechanisms.

To examine whether IDGF2 is able to activate the insulin pathway we compared the ability of IDGF2 and insulin to phosphorylate the insulin downstream targets dS6K (S6 kinase) and dAkt/PKB (protein kinase B). The Cl.8+ cells in MM media poor in nutrients served as a negative control, while the cells in MM plus insulin or in nutrient-rich CM would have their insulin pathway activated. We treated Cl.8+ cells in MM with IDGF2, insulin or IDGF2 plus insulin (70 min or 3 h of exposure) and analysed the extracts by PAGE and western blotting. Cl.8+ cells in CM were used as the control. The western blots were probed with phosphospecific dS6K and dAkt/PKB antibodies. As shown in Fig. 4g,h, the P-Thr-398 dS6K antibody detected the phosphorylated form of dS6K exclusively in the insulin-stimulated cells or the cells in CM, whereas it was almost completely absent in the IDGF2 and control samples (cells starved in MM). Similarly, a high level of the phosphorylated form of dAkt/PKB was observed in insulin-stimulated cells or control cells in CM. The combination of IDGF2 plus insulin produced the same intensity signal for phosphorylated dS6K and dAkt/PKB as insulin alone, suggesting that IDGF2 has no role in the activation of the insulin pathway. Consistently, the pre-treatment of cells with rapamycin (TOR inhibitor) or LY294002 (PI3K inhibitor) abolished dS6K phosphorylation without decreasing the cell viability (Fig. S4).

Our data further confirmed that IDGF2 increases the survival of Cl.8+ cells in MM. The results also reveal that IDGF2 does not activate the insulin pathway. Interestingly, we did not observe any significant effect of insulin on Cl.8+ cell viability under these experimental conditions.

### IDGF2 protects Cl.8+ cells from the deleterious effects of some metabolites and xenobiotics

It has also been shown that IDGF2 promotes the growth of Cl.8+ cells in SFM containing yeast extract^5^. We have previously shown that the proliferation of these cells in such media is blocked by a high level of Ado or dAdo, which are present in the yeast extract, and requires detoxification by bovine adenosine deaminase (ADA) or ADGFs^18^. To test the hypothesis that IDGF2 can protect Cl.8+ cells from high levels of these nucleosides, we examined the effects of Ado and dAdo with and without IDGF2, and assessed cell viability using MTS and TMRE staining. The MTS staining results showed that Ado and dAdo treatment (≥15 μM and ≥70 μM, respectively) caused cell death in MM, while the simultaneous treatment of Cl.8+ cells with 30 μM Ado or 70 μM dAdo together with IDGF2 (16 μg/ml) protected the viability of these cells (Fig. S5a). Similarly, flow-cytometric analysis of TMRE stained cells showed that the Ado treatment increased the number of cells that lost ΔΨm (left peak, Fig. S5d) thus reduced the number of viable cells by about half (right peak), while IDGF2 protected these cells. Consistently, the negative effect of dAdo on ΔΨm was also reduced by IDGF2 (Fig. S5c,e,g,h).

**Figure 5:**
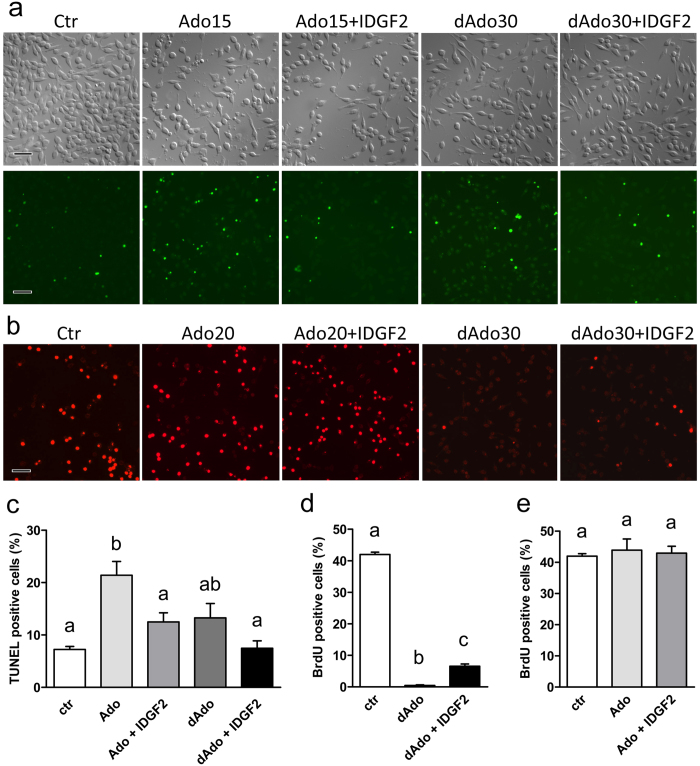
Cytoprotective effects of IDGF2 on Cl.8+ cells in MM treated with Ado and dAdo. (**a**) Effect of Ado (15 μM), IDGF2 (16 μg/μl) plus Ado (15 μM), dAdo (30 μM), and IDGF2 (16 μg/μl) plus dAdo (30 μM) on Cl.8+ cell morphology in the top panels and DNA fragmentation (TUNEL staining) in the bottom panels. Green spots represent the nuclei of apoptotic cells. The addition of IDGF2 reduced the number of apoptotic cells in Ado-treated Cl.8+ cells. Bar = 100 μm. (**b**) BrdU staining (red). The cells were treated with dAdo (30 μM), IDGF2 (16 μg/μl) plus dAdo (30 μM), Ado (20 μM), IDGF2 (16 μg/μl) plus Ado (20 μM). dAdo-treated cells were essentially negative for BrdU incorporation. The addition of IDGF2 to dAdo increased the number of BrdU-positive cells. dAdo treated Cl.8+ cells did not show morphological changes connected with apoptosis, but they did not proliferate. (**c**) Effects of Ado, dAdo and IDGF2 on the relative proportion of the TUNEL-positive (apoptotic cells) Cl.8+ cells. Concentrations: Ado (15 μM), IDGF2 (16 μg/μl), dAdo (30 μM). (**d,e**) Effects of Ado, dAdo and IDGF2 on the relative proportion of the BrdU*-*positive Cl.8+ cells. Concentrations: Ado (20 μM), IDGF2 (16 μg/μl), dAdo (30 μM). Data in the graphs are presented as mean ± SEM (n = 3). Significant differences were evaluated by ANOVA followed by Tukey test and are indicated by different letters (p < 0.05).

The toxicity of Ado and dAdo has previously been reported in other cell types to have different mechanisms of action; while Ado has been implicated in apoptosis by receptor-dependent or independent mechanisms, dAdo has been associated with the block of DNA synthesis followed by progressive cell death^19^. As shown in Fig. 5a, the addition of 15 μM Ado to Cl.8+ cells in MM led to a high proportion of cells with positive TUNEL staining, while co-treatment with IDGF2 brought the rate of TUNEL-positive cells back to normal. Similarly, the BrdU staining showed the inhibition of DNA synthesis at dAdo concentrations of 30 μM and higher, while such cells co-treated with IDGF2 (16 μg/ml) still retained a considerable proportion of cells incorporating BrdU (Fig. 5b). As expected, the effect of 30 μM dAdo on the proportion of TUNEL-positive cells (Fig. 5c) as well as the effect of 20 μM Ado on BrdU-stained cells were not significant (Fig. 5d,e), thus confirming their different mechanisms of action.

Our previous experiments with Ado-treated Cl.8+ cells showed that the excess of extracellular Ado in MM is transported into cells and caused a massive accumulation of ATP followed by the loss of ΔΨm and apoptosis^20^. To test whether IDGF2 influences Ado uptake from the cytoplasm via nucleoside transporters, we compared the uptake of Ado-treated Cl.8+ cells with or without IDGF2. The results showed that IDGF2 had no effect on Ado uptake (Fig. S6a). We also tested whether IDGF2 would decrease the deleterious level of ATP accumulation in Ado-treated Cl.8+ cells (Fig. S6b). Surprisingly, the concentration of ATP was significantly higher in Cl.8+ cells treated with Ado together with IDGF2 than in the Ado-treated controls. However, despite the high level of ATP the IDGF2-treated cells (unlike with the cells treated only with Ado) did not display a dissipation of ΔΨm and survived (Fig. S5e).

**Figure 6:**
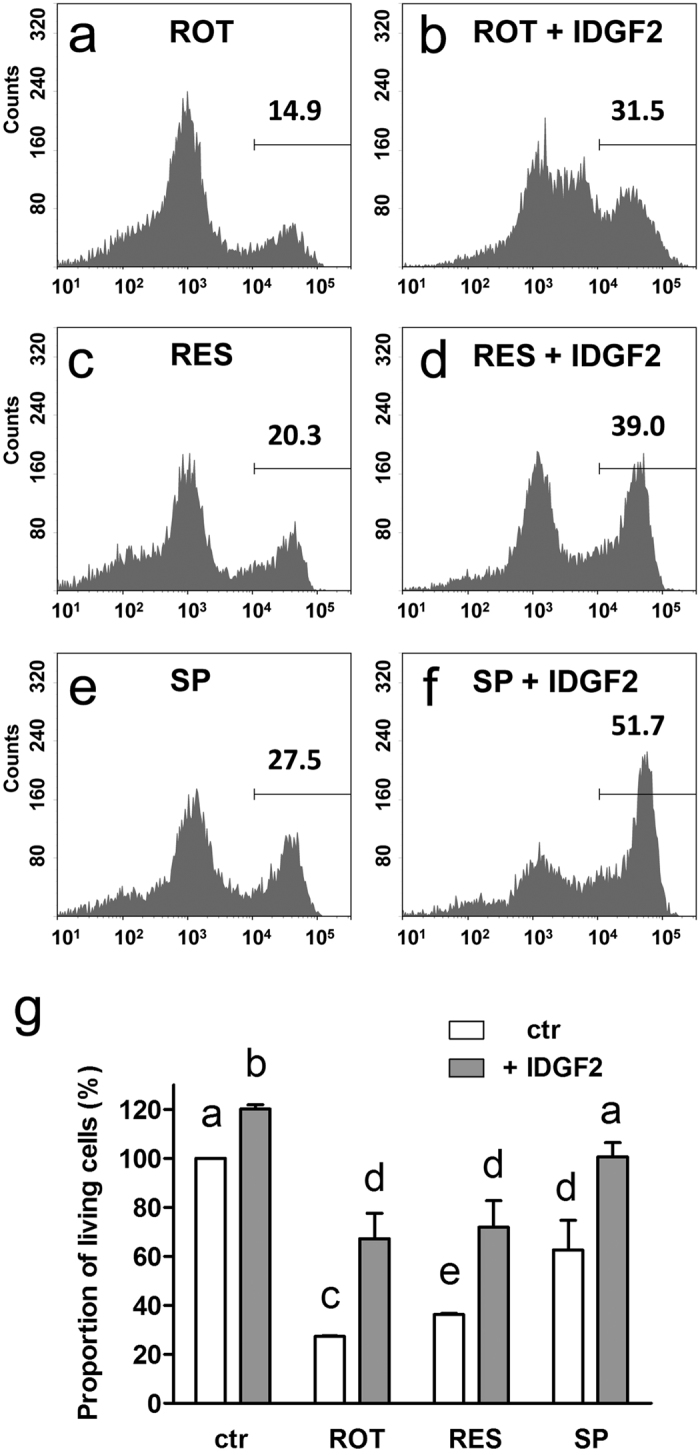
Cytoprotective effects of IDGF2 on Cl.8+ cells treated with xenobiotics. The cells were treated with 0.01 μM rotenone (ROT) (**a,b**), 100 μM resveratrol (RES) (**c,d**), 25 μM SP600125 (SP) (**e,f**) for 16 hrs. Mitochondrial polarity was assessed by flow cytometric analysis of TMRE stained cells. The co-treatment with IDGF2 (16 μg/ml) partially rescued cell viability (**b**,**d**,**f**). (**g**) Effects of IDGF2 on the viability of cells treated by xenobiotics. The graph summarizes three cell cytometry experiments. Data in the graphs are presented as mean ± SEM. Significant differences were evaluated by ANOVA followed by Tukey test and are indicated by different letters (p < 0.05).

To examine, whether IDGF2 could act as a protective agent against more types of toxic substances, we tested three xenobiotics, which have previously been studied in *Drosophila*, including rotenone (an inhibitor of mitochondrial respiratory complex I)^21^, resveratrol (an AMPK activator that confers cytoprotection in various models of oxidative injury)^22^ and SP600125 (a JNK inhibitor)^23^. We assessed the toxic effects of these chemicals and chose the concentrations, close to the threshold dose levels at which toxicity first appears. The Cl.8+ cells with or without IDGF2 (16 μg/ml) co-treatment were incubated in MM with the respective xenobiotic for 16 hrs. The resulting cell viability was assessed by TMRE staining and flow cytometry. As shown in Fig. 6, the co-treatment of Cl.8+ cells with IDGF2 dramatically reduced the effect of examined xenobiotics on ΔΨm.

Interestingly, we observed the cytoprotective effects of IDGF2 on Cl.8+ cells treated with the xenobiotics or metabolites only in minimal media, but not in cells cultured under more physiological conditions in complete culture media containing FBS, fly extracts and yeast extracts. For comparison we also tested the effects of Ado and xenobiotics in MM supplemented with 2% FBS, 2.5% fly extracts, or both (Fig. S7). The results show that FBS and partly also fly extract stabilize ΔΨm to a similar extent and reduce the toxicity of low doses of xenobiotics and metabolites. It is not clear whether FBS and IDGF2 work via similar mechanisms (fly extracts contain a mix of IDGF proteins).

Taken together, our results further suggest that IDGF2 effectively stabilizes ΔΨm and inhibits the ΔΨm dissipation induced by a wide range of agents. The cytoprotective effect of IDGF2 was shown to be of a similar extent to that of FBS.

### Downstream targets of IDGF2

To better understand the mechanism of IDGF2 action, we performed genome-wide transcriptional analysis to identify genes that were differentially expressed in Cl.8+ cells in response to IDGF2. Treatment of Cl.8+ cells with IDGF2 altered the expression of 81 genes, which passed our filtering criteria (p-value < 0.05 and |logFC| > 0.8) for statistical significance (Table S2). Microarray data classification based on functional groupings (gene ontology, GO) showed that most of the induced genes are involved in innate immunity (22.2%), morphogenesis (22.2%), metabolic process (14.8%), various transporters (14.8%), cytoskeleton organization (11%) and response to stimulus (10%) (Table S2). Interestingly, at least seven of the induced genes (8.6%) were implicated earlier in the response to xenobiotics^24^.

**Figure 7:**
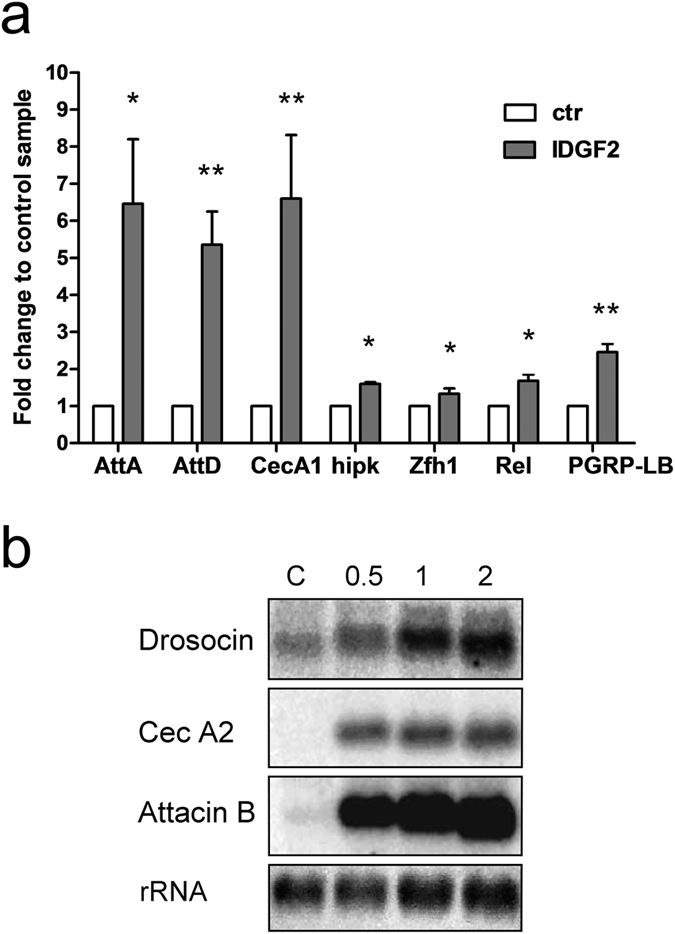
Validation of microarray gene expression changes induced by IDGF2 in Cl.8+ cells. (**a**) The Cl.8+ cells were induced by the addition of IDGF2 (16 μg/ml) and relative changes in gene expression were analysed by real-time reverse transcription PCR (RNA was isolated 4 h after treatment). n = 3. *AttA* - *Attacin A; AttD - Attacin D; CecA1 - Cecropin A1; hipk - Homeodomain interacting protein kinase; Zfh1 - Zn finger homeodomain 1; Rel – Relish; PGRP-LB - Peptidoglycan recognition protein LB.* Data in the graph are presented as mean ± SEM (n = 3). Significant differences (*p < 0.05, **p < 0.01) between control and IDGF2 treatment are indicated by asterisks and were evaluated by Student’s t-test. (**b**) *Drosocin (CG10816*), *cecropin A2 (CG1367*) and *attacin B (CG18372*) transcripts in IDGF2-treated Cl.8+ cells were examined by northern analysis. Numbers on top denote time intervals following IDGF2 addition in h. The bottom panel shows methylene blue-stained rRNA as a loading control. Full-length blots are shown in Fig. S11.

We verified the IDGF2-mediated upregulation of several genes in Cl.8+ cells using real-time RT-PCR. They included *Attacin A* and *D, Cecropin A1, PGRP-LB* and *Rel* from the *Imd* immune pathway and *Homeodomain interacting protein kinase (hipk*) from the *Wnt* pathway, as well as *Zfh1* implicated in the activation of nephrocytes (Fig. 7a). In addition, the kinetics of induction of several IDGF2-inducible immune genes, including *Drosocin, Cecropin A2* and *Attacin B* was examined by northern blot analysis. As shown in Fig. 7b, the induction of these transcripts occurred within 30 min after IDGF treatment, suggesting that they represent the primary response genes.

For comparison, we extended our microarray analysis and included the expression profiles of Cl.8+ cells treated with the combination of IDGF2 (16 μg/ml) and 50 μM Ado as well as with 50 μM Ado only. The treatment with a combination of IDGF2 and Ado significantly changed the transcript levels of 196 genes while the treatment with Ado-only altered the expression rates of 117 genes fulfilling our filtering criteria (p-value < 0.05 and |logFC| > 0.8). The proportions of genes in functional GO groups are shown in Tables S3 and S4.

To compare the lists of differentially expressed genes for the three treatments we constructed a Venn diagram (Fig. S8). We inspected in more detail the genes expressed exclusively in the cells simultaneously treated with Ado+IDGF2, since they might be involved in the compensatory homeostatic mechanisms allowing the survival of Ado-treated Cl.8+ cells. Candidate genes for such compensatory changes may include the upregulated trehalose transporters *Tret1-1* and *Tret1-2* or predicted sugar transporter-like gene *CG3168,* presumably needed for energy mobilization. Among other candidates, which may also influence energy homeostasis, we noticed downregulated *ATPase (V0 complex)* and upregulated *5*′*-nucleotidase CG32549* (Table S4).

The results of gene-enrichment analysis are shown in Table S5. The major significantly enriched KEGG pathway of differentially expressed genes for IDGF2 is a phagosome pathway, which is an important counterpart of the defence mechanisms. In contrast, Ado treatment had the largest impact on the downregulation of pantothenate and CoA biosynthesis, key precursors required for many biosynthetic reactions involved in lipogenesis^25^. Interestingly, the simultaneous treatment of Cl.8+ cells with Ado+IDGF2 lead to the strong upregulation of three overlapping pathways involved in organismal detoxification containing glutathione as well as cytochrome P450 gene family members (Table S5).

Our data suggest that IDGF2 is involved in several biological processes. The IDGF2 treatment of Cl.8+ cells activates the immune response and its component phagocytosis as indicated by GO annotation and GSEA-KEGG analyses, respectively. Interestingly, the simultaneous treatment of Cl.8+ cells with IDGF2 and Ado lead to the up-regulation of multiple genes involved in stress and detoxification as well as energy metabolism.

### IDGF2 is localized in pericardial and garland cells *in vivo* and is induced by aseptic or septic injury

**Figure 8:**
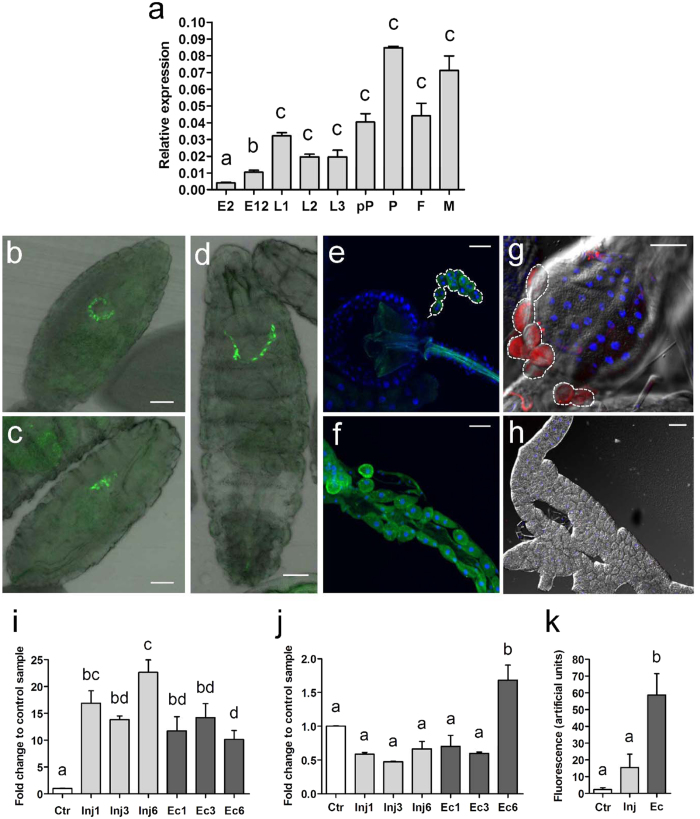
Expression and localization profile of IDGF2 in development and by septic and aseptic injury. (**a**) Developmental expression profile of *Idgf2* assayed by qRT-PCR. E2 - embryos 2 h, E12 - embryos 12 h, L1 - first instar larvae, L2 - second instar larvae, L3 - third instar larvae, pP - white prepupae, P - pupae (stage P10), F - females, M - males. rp49 mRNA was used as an internal control. Data in the graphs are presented as mean ± SEM (n = 3). (**b–f**) Localization of IDGF2::GFP (green) driven by the IDGF2 promoter. (**b,c**) - embryo, stage 16, dorsal and lateral view, (**d**) - first instar larva, (**e**) - proventriculus with attached garland cells (tracing line) from third instar larva, (**f**) - aorta from third instar larva. IDGF2::GFP fusion protein was detected by anti*-*GFP antibody. Cell nuclei were stained with DAPI (blue). Scale bar 50 μm. IDGF2 protein was mostly localized in garland cells (**b–e**) and pericardial cells (**f**). (**g–h**) IDGF2-myc was expressed in the fat body (by *UAS-Gal4* system) and detected with anti-myc antibody (red). Strongest IDGF2-myc signal was accumulated in garland cells (tracing line), scale bar 50 μm (**g**). While almost no signal was observed in the fat body, scale bar 100 μm (**h**). *Idgf2* expression change in third instar larvae (**i**) and imagoes (**j**) after aseptic (light gray columns) or septic injury (dark gray columns) with *E. coli*. Non-wounded larvae and flies served as controls. In both sets of experiments, total RNA was extracted 1, 3 and 6 h after injury from whole animals. *rp49* mRNA was used as an internal control (n = 3). (**k**) Fluorescence signal from Idgf2::GFP in garland cells in L3 larva responds to infection after aseptic (light gray columns) or septic injury (dark gray columns) with *E. coli*. The values were derived from fluorescence images using the ImageJ analysis program. Non-wounded larvae served as controls (n = 4). Data in the graphs are presented as mean ± SEM. Significant differences were evaluated by ANOVA followed by Tukey test and are indicated by different letters (p < 0.05).

In order to obtain information on the function of IDGF2 *in vivo*, we examined the developmental expression patterns of IDGF2 by qPCR. The results showed that IDGF2 is expressed at all stages assessed, and the highest expression levels were detected in pupae and adult males (Fig. 8a).

The tissue distribution of the IDGF2 transcript in *Drosophila* larvae was examined by *in situ* hybridization, and confirmed the fat body as major sites of IDGF2 expression^5^ (Fig. S9a). We also examined the IDGF2 protein distribution in *Drosophila* larvae of first and third instar using transgenic flies carrying the *Idgf2::GFP* construct and immunostaining with an anti-GFP antibody. As shown in Fig. 8b–f, a high amount of IDGF2 protein was present in garland and pericardial cells, *Drosophila* nephrocytes that are an important part of the excretory system and are implicated in the detoxification of the insect body^26^. Interestingly, we did not detect any *Idgf2* transcripts in garland cells by *in situ* hybridization (Fig. S9c), suggesting that proteins accumulate in garland cells after being secreted into the haemolymph. To verify this idea, we also expressed the *UAS-IDGF2-myc* transgene under the fat body-specific driver (*Lsp2-Gal4*) and examined the localization of the recombinant IDGF2. While the strongest IDGF2-myc signal detected with anti-myc antibody was again observed in garland cells (Fig. 8g), the fat body contained almost no signal (Fig. 8h) probably because of its rapid release into the haemolymph.

Since various CLPs were earlier implicated in responses to bacteria and tissue remodelling^27^, we tested *Idgf2* expression in response to aseptic as well as septic injury in third instar larvae and adult male flies. Septic injury was performed with a syringe needle dipped in a culture of Gram-negative bacteria (*E. coli*). Changes in relative *Idgf2* transcript levels were determined 1, 3 and 6 h after the injury by qRT-PCR. The results confirmed that *Idgf2* expression is induced after both septic and aseptic injury in the larval stage but not in adults, except for a modest induction observed 6 h after septic injury. Interestingly, in larvae, *Idgf2* mRNA induction after 6 h was greater in the aseptic injury experiment than under septic conditions (Fig. 8i,j). In contrast, the staining of IDGF2 in larval garland cells increased dramatically after septic injury (Fig. 8k).

Our results show that IDGF2 is activated by septic and aseptic injury at the larval stage and to some extent also in adults. The accumulation of IDGF2 in garland and pericardial nephrocytes is consistent with its role in organismal detoxification.

## Discussion

IDGF2 seems to be an unusual invertebrate mitogen from several points of view. It was discovered in the conditioned media of *Drosophila* cells, it is present in *Drosophila* haemolymph in high concentrations, and it has a carbohydrate-binding capacity. IDGF2 has been reported earlier to have a synergistic effect with insulin, potentially functioning as its cofactor^5^. However, our results show that IDGF2 does not act through the *Drosophila* insulin receptor, and that it has a different effect on cells. Taken together, our data strongly suggest that IDGF2 and insulin activate different pathways, however, we cannot exclude that some of the native *Drosophila* insulin-like peptides play a cooperative or moderating role on IDGF2 *in vivo*. The relatively weak effect of insulin on Cl.8+ cell proliferation in Shields and Sang M3-based growth medium has previously been observed and ascribed to the specific composition of this medium^28^.

The cytoprotective function of IDGF2 is best seen in Cl.8+ cells, but we also observed the subtle effects of IDGF2 on other cell types, including the embryonic cell line S2 (Fig. S10). IDGF2 enhances the survival of Cl.8+ cells in serum-free conditions of SFM or MM, but it shows only a marginal effect in CM (Fig. S2). Despite of the high concentration of IDGF2 used in our experiments only relatively few cells show a response. For example, only about 30% of Cl.8+ cells survived and continued to divide in SFM at physiological IDGF2 concentration (Fig. 3). The variability in the IDGF2 response did not seem to be caused by the potential heterogeneity of Cl.8+ cells. The cell survival seemed to be a stochastic process, and the surviving cells did not become more resistant to Ado or to other deleterious conditions. The effect of IDGF2 rather suggested that it can renew homeostatic equilibrium only in part of the cell population.

IDGF2-induced cytoprotection seems to cover a wide range of harmful conditions and may involve several mechanisms. IDGF2 enhanced the survival of Cl.8+ cells in the serum-free and low nutrient MM conditions as well as protecting them from cytotoxic levels of Ado, dAdo and certain xenobiotics. Interestingly, it has previously been suggested that the cytotoxic effects of some treatments and chemicals disappear in the presence of serum proteins, which keep the cells in a more physiological state^29^. We found that IDGF2 affected Cl.8+ cells in a similar way to FBS, with the 2% FBS showing similar cytoprotective effects on the toxicity of Ado and xenobiotics. However, in contrast to IDGF2, FBS was also shown to have some adenosine deaminase activity, specific to the detoxification of Ado and dAdo^18^. FBS is routinely added to insect culture media at similar concentrations as in vertebrate cell cultures^30^, but its role is poorly understood. Our data suggest that Cl.8+ cells require the binding of IDGF2 for their proliferation, and FBS may be a substitute for this requirement.

Our differential expression profiling of genes involved in IDGF2-induced cytoprotection suggested that the Cl.8+ cell response against Ado involves the mobilization of energy-providing carbohydrates as well as the adjustment of purine metabolism. We have previously reported that Ado treatment caused the excessive uptake and recycling of extracellular Ado and a subsequent high ATP production and followed by the loss of ΔΨm, which were considered to be hallmarks of Cl.8+ cell apoptosis^20^. Here, we show that the Cl.8+ cells treated simultaneously with Ado and IDGF2 reach even higher ATP levels than do cells treated only with Ado, but that such an increase was not accompanied by the loss of ΔΨm and cell death. It therefore seems that the depolarization of ΔΨm in Cl.8+ cells is a key event for the initiation of Ado-induced apoptosis and IDGF2 might be able to renew the mitochondrial and cellular energy homeostasis. Interestingly, earlier reports on mouse cells showed that the increase of cytosolic Ado affects mitochondrial adenosine production and disrupts mitochondrial bioenergetics^31^.

Our results also revealed that IDGF2 is able to increase BrdU incorporation in dAdo-treated cells (Fig. 5b). Since it has been earlier reported that, in mammalian cells, the dAdo toxicity is caused by the imbalance in purine metabolism leading to the block of DNA synthesis and cell death^19^, it seems that the IDGF2-induced cytoprotection against dAdo toxicity in Cl.8+ cells might also be connected with homeostasis in the purine metabolism.

Alternatively, the cytoprotective effects of IDGF2 might be related to the regulation of detoxification. Our transcriptional profiling of Cl.8+ cells simultaneously treated with IDGF2 and Ado revealed the induction of a number of detoxification enzymes, including glutathione-S-transferases and members of the cytochrome P450 family. We suggest that the induction of detoxification mechanisms may play important role, especially in the protection of Cl.8+ cells from the toxicity of some xenobiotics.

Our experiments indicated that IDGF2 also plays protective roles at the level of the organism. Our expression profiling analysis revealed the activation of genes involved in organismal detoxification including the genes specific to the function of *Drosophila* nephrocytes (garland and pericardial cells)^32^, such as *Zn finger homeodomain 1* (involved in the differentiation of garland cells) and *Mec2* (a podocin homolog involved in nephrocyte filtration). This observation is further supported by the *in vivo* localization of IDGF2 in garland and pericardial cells.

Moreover, IDGF2 might also have an important protective immune function. It was shown for the closely related IDGF3 protein that *Drosophila IDGF3* mutants are more susceptible to infection with the nematodes or Gram-negative bacteria^27^. IDGF1 and 3 have been previously suggested as pattern recognition molecules^33^ able to recognize chitin or related carbohydrates on the surface of nematodes and other parasites and thus activate immune effector mechanisms^27^. We observed IDGF2 induction by aseptic or septic injury and confirmed its ability to induce the transcription of a number of defence-related genes, including the key NF-κB transcription factor *relish*^34^ and many antibacterial peptides (Fig. 7a,b). IDGF2 might be involved in wound healing rather than in the response to infection, since the *Idgf2* induction after 6 h was greater in the aseptic injury experiment than under septic conditions (Fig. 8i).

Taken together, there is an interesting parallel between the effects of IDGF2 and some mammalian members of the 18 glycosyl hydrolase family. The prototypical YKL-40 protein has been reported in normal human serum at concentrations in the nanomolar range and is increased in the serum of patients with arthritis or asthma^35^. Recent reports have demonstrated the role of YKL-40 in diabetes and the promotion of tumour expansion^36^,^37^. This all suggests that vertebrate and insect CLPs are involved in similar cellular and organismal functions; they bind to the carbohydrate components of a number of cell surface receptors and regulate multiple signalling pathways^10^,^11^,^12^. Thus, further investigation of the downstream signalling pathways and exact biological function of IDGF2 and other CLPs require more studies. IDGF2 can serve as a suitable model for the research on other CLPs.

In summary, our research fills an important gap in the knowledge of *Drosophila* growth factors. We show that IDGF2 does not activate the insulin pathway but rather is involved in the enhancement of cellular survival under starvation conditions, and elevated levels of extracellular Ado, dAdo and xenobiotics *in vitro*. It seems that the IDGF2 keeps the cells in more physiological state, so that they can devote more resources to the detoxification or elimination of a toxic agent or for the renewal of homeostasis. *Drosophila* IDGF2 is an important trophic factor with similar overall homeostatic effect as mammalian serum proteins. Recombinant IDGF2 may serve as an important additive to *in vitro* cell and organ cultures.

## Materials and Methods

### Drosophila stocks

All flies were reared at 25 °C on a standard cornmeal-yeast-agar diet. Oregon-R flies (from The Bloomington *Drosophila* Stock Center, BDSC) were used for the determination of *Idgf2* expression profile and injury experiments. Transgenic flies carrying *Idgf2::GFP* constructs were generated as described earlier^27^. The oligonucleotides containing 50-bp homology arms for recombineering followed by the linker region of amplification are shown in Table S1. For generation of *UAS*-*Idgf2-myc* fly strains we followed the protocol published previously^27^. cDNA from *Idgf2* was amplified from EST-clone *GH12581* by primers listed in Table S1. Stable stocks with *UAS*-*Idgf2-myc* were produced using standard P-element transformation of the w^11^,^18^ flies. A homozygous-viable double insertion on the 2nd and 3rd chromosome was used for further experiments together with *Lsp2*-*Gal4* (BDSC).

### Recombinant protein isolation

The Bac-to-Bac (Invitrogen) method was used for the production of histidine-tagged recombinant IDGF2. First, *Idgf2* cDNA was subcloned into the transfer vector and used for recombination with baculovirus DNA according to the manufacturer’s instructions. Sf9 cells were used for transfections and virus amplification. Hi5 cells, used for protein production, were infected with a multiplicity of infection of 10 plaque-forming units (pfu) per cell. Three days post-infection, the medium was harvested, dialyzed against binding buffer and used for protein purification. Recombinant proteins were purified using a Ni-nitrilotriacetic acid (NTA) column (Qiagen). Fractions containing recombinant proteins were pooled and dialyzed in PBS. The IDGF2 concentrations were determined by Bradford assay (Sigma-Aldrich).

### Cell culture

The *Drosophila* imaginal disc cell line Cl.8+ 17 was grown at 25 °C in complete medium (CM) containing Shields and Sang M3 Insect Medium (Sigma-Aldrich) supplemented with 2% fetal bovine serum (FBS), 2.5% fly extract, and 0.125 IU/mL insulin. The “supplement-free medium” (SFM) was Shields and Sang medium (Sigma-Aldrich, S8398) containing yeast extract (1 g/l). The “minimal medium” (MM) was Shields and Sang medium (Sigma-Aldrich, S8523) lacking yeast extract, supplemented with L-leucine, L-methionine, fumaric acid, folic acid, myo-inositol, pyridoxine, riboflavin, thiamine and insulin (0.125 IU/mL). Since this medium was fully chemically defined, it was used in most experiments. Embryonic S2 cells were maintained in Shields and Sang medium supplemented with 10% FBS.

Lepidopteran cells Sf9 and Hi5 used for baculovirus expression were maintained at 27 °C in TNM-FH medium (Sigma-Aldrich) supplemented with 10% FBS. Hi5 cells were maintained in EX-CELL Serum-Free medium (Sigma-Aldrich).

### Functional *in vitro* assays

Cell viability was assessed using the CellTiter 96 AQueous One Solution cell kit (Promega), which contains a tetrazolium compound MTS. Cl.8+ cells were seeded at a concentration of 1 × 10^6^/ml in a flat-bottomed 96-well plate in minimal medium (MM) with or without recombinant IDGF2 (16 μg/ml), Ado and dAdo and incubated for 22 h. Next, 20 μl of MTS solution was added to each well and the plate was incubated for an additional 3 h at 25 °C. The absorbance at 490 nm was recorded with a 96-well plate reader. The assay was performed with five replicates for each sample.

Proliferation of Cl.8+ cells in SFM and CM was also measured by the direct counting of cells using digital photographs of identical areas (0.8 × 0.8 mm) taken every 24 hrs. Each value represents an average of three fields per plate.

TUNEL staining, BrdU incorporation, the ATP assay and the Ado uptake assay were performed as described previously^20^. For TUNEL staining, we used the *In Situ* Cell Death Detection kit with fluorescein (Roche), and for BrdU incorporation, we used the *In Situ* Cell Proliferation Kit, FLUOS (Roche). ATP was measured by The CellTiter-Glo^®^ Luminescent Cell Viability Assay (Promega). The Ado uptake assay was performed using H^3^-Ado in MM.

### Cell cytometry

The ΔΨm analysis is based on the property of living cells with active mitochondria to sequester the TMRE stain (right peaks), while TMRE-negative cells lose their ΔΨm and undergo apoptosis (left peaks). To stain for mitochondrial polarity, Cl.8+ cells were incubated with 600 nM tetramethyl rhodamine ethyl ester (TMRE, Molecular Probes), for 30 min at 25 °C in culture medium. The dye was taken up by mitochondria and mitochondrial staining was analysed by flow cytometry in the 585/40 channel using ACEA NovoCyte^TM^ 3000. At least 12,000 events were used for analysis. Morphological analysis is based on the measurement of cell size and granularity as indicated by the flow cytometry forward scatter versus side scatter (FSC/SSC) intensity plots.

The Cl.8+ cells were grown in 12-well plates at a concentration of 5 × 10^5^/ml in MM or CM and treated with recombinant IDGF2 (16 μg/ml), Ado (30–100 μM) and dAdo (70–100 μM). Rotenone (0.01 μM, Sigma-Aldrich), resveratrol (100 μM, Tocris Bioscience) and SP600125 (25 μM, Tocris Bioscience) were used at the threshold dose level at which the toxicity was low but detectable. In all these experiments, the same volumes of the corresponding solvents were used in the controls.

### Western blotting and immunostaining

For IDGF2 protein quantification, hemolymph collected from wandering third instar larvae or known amounts of recombinant IDGF2 were subjected to SDS PAGE. SDS-PAGE and western blotting were performed as described earlier^38^. Samples of recombinant IDGF2 were electrophoresed in parallel. Proteins were transferred to PVDF membranes and detected using an antibody against IDGF2. The anti-IDGF2 rabbit polyclonal antibody was developed using a KLH-coupled synthetic peptide IYHPDGSKDRLAH (Genscript). HRP-conjugated goat anti-rabbit IgG were used as the secondary antibody (1:5000, Jackson Immunoresearch). The immunocomplexes were detected using a chemiluminescence reagent kit (Thermo Scientific). Protein bands were quantified with a LAS-3000 imaging system (Fuji Corp.). A standard curve was constructed from the readouts of recombinant IDGF2 protein. The resulting value was obtained by averaging the data from three independent experiments.

For the dS6K and dAkt/PKB phosphorylation assay, Cl.8+ cells were grown in 12-well plates at a concentration of 5 × 10^5^/ml, starved (in a 4:1 dilution ratio of MM to CM media) for 24 h and treated with recombinant IDGF2 (16 μg/ml) or insulin (0.125 IU/mL) for 70 min or 3 h in MM. The cells were also pretreated for 30 min with 15 μM LY294002 (PI3K inhibitor) or for 15 min with 20 nM rapamycin (TOR inhibitor). The rabbit anti-phospho-dS6K (Thr398) and anti-phospho-dAkt/PKB (S505) antibodies were from Cell Signaling Technology (1:1000); the monoclonal anti-α-tubulin antibody (T9026) was from Sigma-Aldrich (1:2000).

A rabbit polyclonal anti-GFP (1:500 dilution, Invitrogen) antibody was used to detect the IDGF2::GFP fusion protein, with subsequent detection using an AlexaFluor 488-conjugated anti-rabbit antibody (1:2000 dilution, Abcam). IDGF2-myc was detected with mouse anti-myc antibody (1:500 dilution, Sigma Aldrich) and goat anti-mouse antibody conjugated with Cy3 (1:2000 dilution, Jackson Immunoresearch). DAPI (1:1000, Sigma-Aldrich) was used for the visualization of cell nuclei. Fluorescent images were acquired using a confocal microscope (Olympus Fluoview FV1000). Images were analysed with the ImageJ graphics program.

### Northern blotting and microarray analysis

Northern blotting was performed as described earlier^38^. Total RNA was isolated from control and IDGF2-treated cells (16 µg/ml for 4 h). using TRIZOL (Life Technologies). Cl.8+ cells were lysed in TRIZOL and the aqueous phase was used for further purification on RNeasy spin columns (Qiagen RNeasy kit).

The Affymetrix GeneChip^®^ Drosophila Genome Array System was used for the microarray analysis following the standard protocol (100 ng RNA was amplified with GeneChip 3′ IVT Express Kit (Affymetrix) and 10 μg of labeled cRNA was hybridized onto the chip according to the protocols provided by the manufacturer. Labelled cDNA was hybridized to an Affymetrix GeneChip Drosophila Genome Array. The analysis was performed using three replicates. Data were pre-processed in the Partek Genomic Suite (Partek Incorporated), as described previously^39^ and various Bioconductor packages, including Limma^40^. In short, the transcription profiles were background corrected using the GCRMA method, quantile normalized and variance stabilized using the base-2 logarithmic transformation. Limma was used for differential expression analysis. Deregulated genes were selected based on FDR (false discovery rate) <0.05. FDR was calculated by BH (Benjamini-Hochberg) procedure. The transcription data are MIAME compliant and deposited in the ArrayExpress database (accession E-MTAB-5007). All statistical analyses were performed in R software (http://www.R-project.org) via Bioconductor. Gene set enrichment analysis and determination of gene functions were performed using DAVID Bioinformatics Resources web service^41^ and AmiGO2^42^.

### Real-Time RT-PCR

Total RNA isolation, subsequent reverse transcription and real-time RT-PCR from different time points of *Drosophila* development or from larvae with septic or aseptic injury was performed as described previously^27^. For real-time RT-PCR from Cl.8+ cells, we followed a protocol described previously^20^. The primers are shown in Table S1.

### *Drosophila* septic and aseptic injury assays

Injury experiments were performed according to DeGregorio *et al*.^43^. We used the wandering third instar larvae or 3 day old adult males. The aseptic injury was performed by pricking the larvae or fly thorax with a sterile 0.2 mm syringe needle, while for septic injury the needle was first dipped into a concentrated *Escherichia coli* K12 culture. The puncture wounds were small, and no discernible amount of body fluid was lost from the injured animals. The flies were then kept at 25 °C and collected at specific time points after injury (1, 3 and 6 h).

## Additional Information

**How to cite this article:** Broz, V. *et al*. *Drosophila* imaginal disc growth factor 2 is a trophic factor involved in energy balance, detoxification, and innate immunity. *Sci. Rep.* **7**, 43273; doi: 10.1038/srep43273 (2017).

**Publisher's note:** Springer Nature remains neutral with regard to jurisdictional claims in published maps and institutional affiliations.

## Supplementary Material

Supplementary Information
