# Supplementary material for: Drosophila imaginal disc growth factor 2 is a trophic factor involved in energy balance, detoxification, and innate immunity
Source: Sci Rep. 2017 Feb 23;7:43273. doi: 10.1038/srep43273 (PMC5322392; doi:10.1038/srep43273)
Supplement: Supplementary Information [file srep43273-s1.doc]

***Drosophila* imaginal disc growth factor 2 is a trophic factor involved in energy balance, detoxification, and innate immunity**

Vaclav Broz^1,2^, Lucie Kucerova^1^, Lenka Rouhova^2^, Jana Fleischmannova^1^, Hynek Strnad^3^, Peter J. Bryant^4^ and Michal Zurovec^1,2^

^1^Institute of Entomology, Biology Centre CAS, Branisovska 31, 370 05 Ceske Budejovice, Czech Republic;

^2^Faculty of Science, University of South Bohemia, Branisovska 31, 370 05 Ceske Budejovice, Czech Republic;

^3^Institute of Molecular Genetics CAS, Videnska 1083, 142 20 Prague 4; Czech Republic;

^4^Developmental & Cell Biology, School of Biological Sciences, University of California, Irvine, USA.

**Figure S1**. **Dose effect of IDGF2 treatment on *Drosophila* Cl.8+ cells**. Histograms (**a-n**) and FSC/SSC plots (**a´-n´**) show mitochondrial polarity assessed by flow cytometric analysis of TMRE stained cells in SFM (**a-g**) and chemically defined media MM (**h-n**). The cells with active mitochondria (sequestering TMRE) correspond to right peaks in histograms (a-n) and to pink cell populations shown in scatter plots (**a´-n´**). The left peaks in histograms (a-n) and orange cell populations in scatter plots (**a´-n´**) consist of TMRE-negative cells undergoing apoptosis. Apoptotic cells are clearly distinguishable from normal Cl.8+ cells in scatter plots by having reduced cell size (low FSC) and enhanced density (high SSC).

*
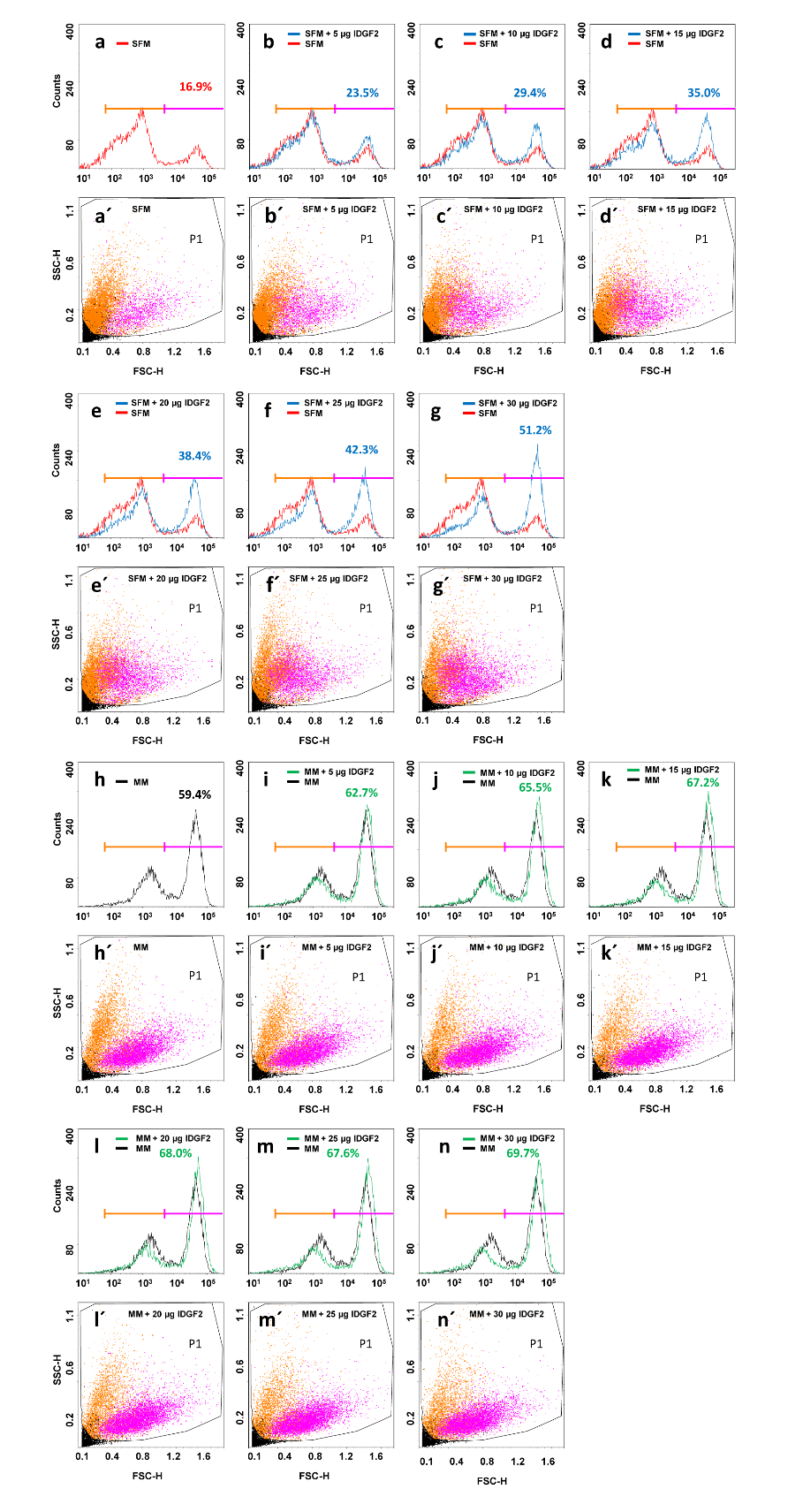
*

**Figure S2**. **IDGF2 has only a marginal effect on Cl.8+ in the complete media (CM).** Mitochondrial polarity was assessed by flow cytometric analysis of TMRE stained cells. Numbers represent the proportion of viable cells.

*
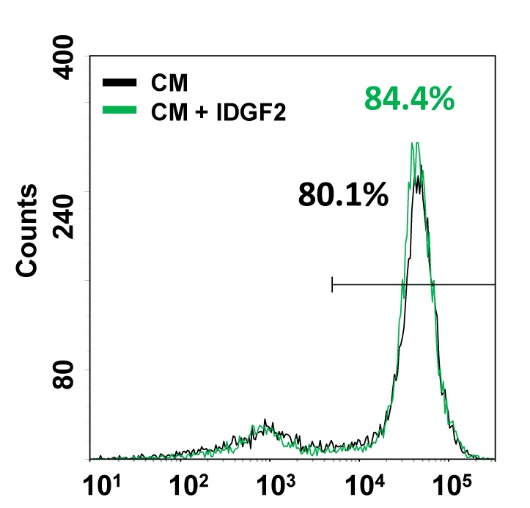
*

**Figure S3**. **Results of three separate experiments comparing the effects of insulin and IDGF2 on Cl.8+ cells in chemically defined media**. The figure shows histograms of mitochondrial membrane potential assessed by TMRE fluorescence. The cells were analyzed 24 h after the transfer from CM to MM (black) or to MM supplemented with insulin (red), IDGF2 (green) or both (blue). Numbers represent the proportion of viable cells. IDGF2 (16 μg/ml) treated cells had a higher proportion of living cells (panels C, G and K compared to the TMRE-control cells). Insulin treatment (0.125 IU/ml) led to a shift of the right peak further to the right (panels B, F and J), suggesting an increase in ΔΨm.

*
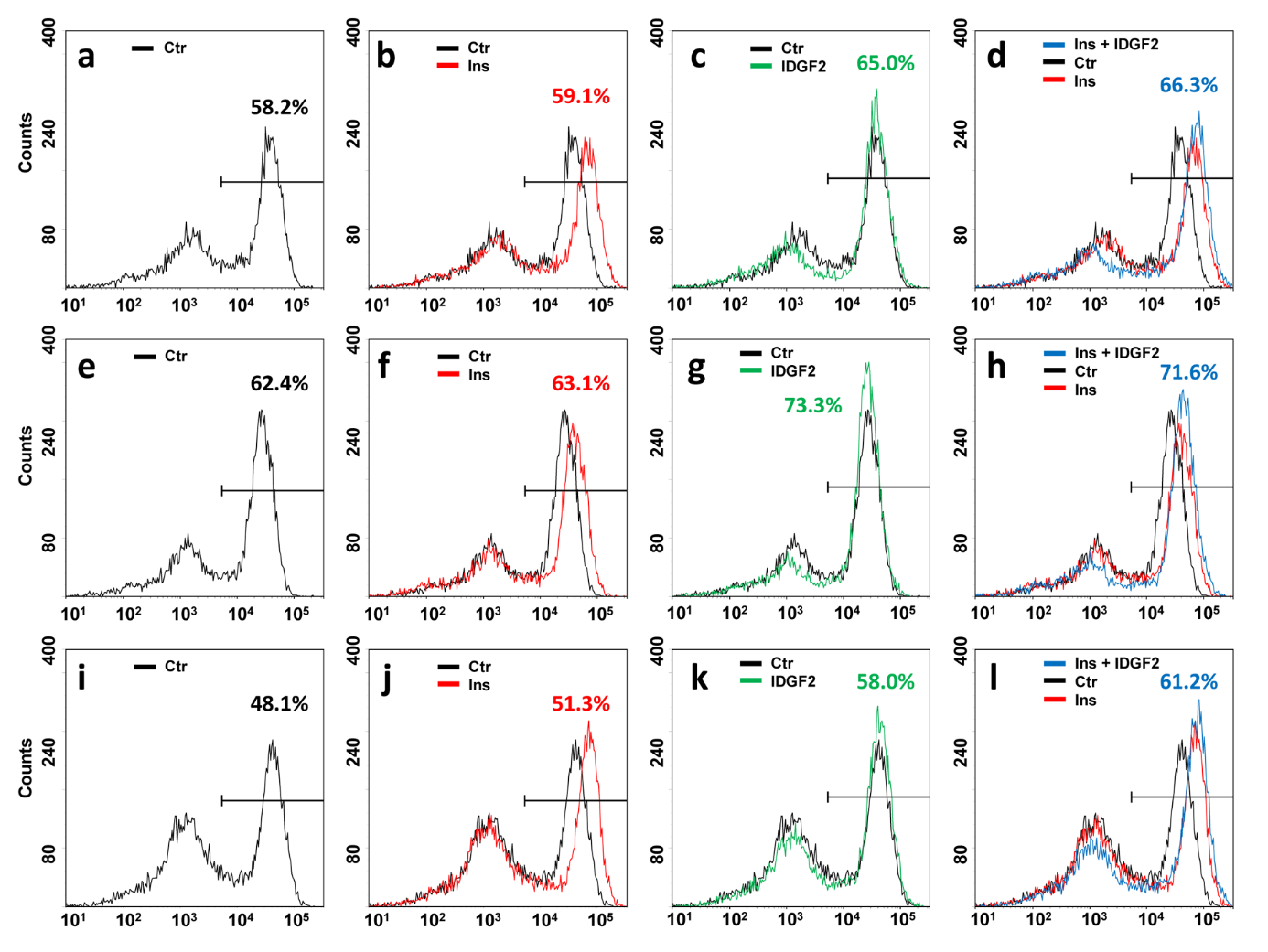
*

**Figure S4**. **Western blot analysis comparing the levels of phosphorylated dS6K (T398) in Cl.8+ cells pretreated with Rapamycin or LY294002**. (**a**) Rapamycin (20 nM) and LY294002 (15 µM) abolished dS6K phosphorylation. Both drugs did not affect cell viability determined by TMRE staining and flow cytometry (c,d). The cells were treated for 16 h in MM (**b**); MM + 20 nM rapamycin (**c**) and MM + 15 µM LY294002 (**d**). Numbers represent the proportion of viable cells.

*
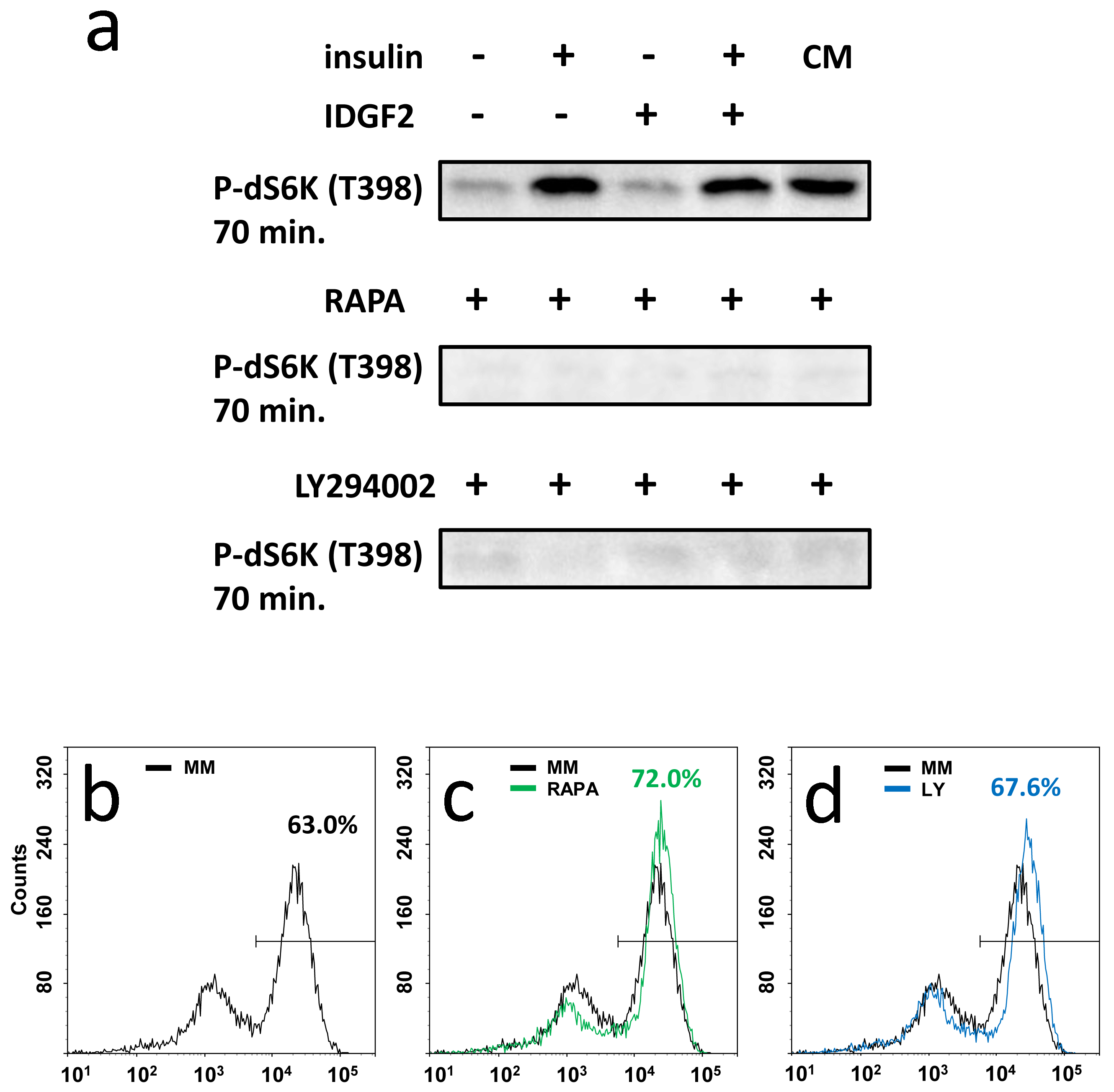
*

***
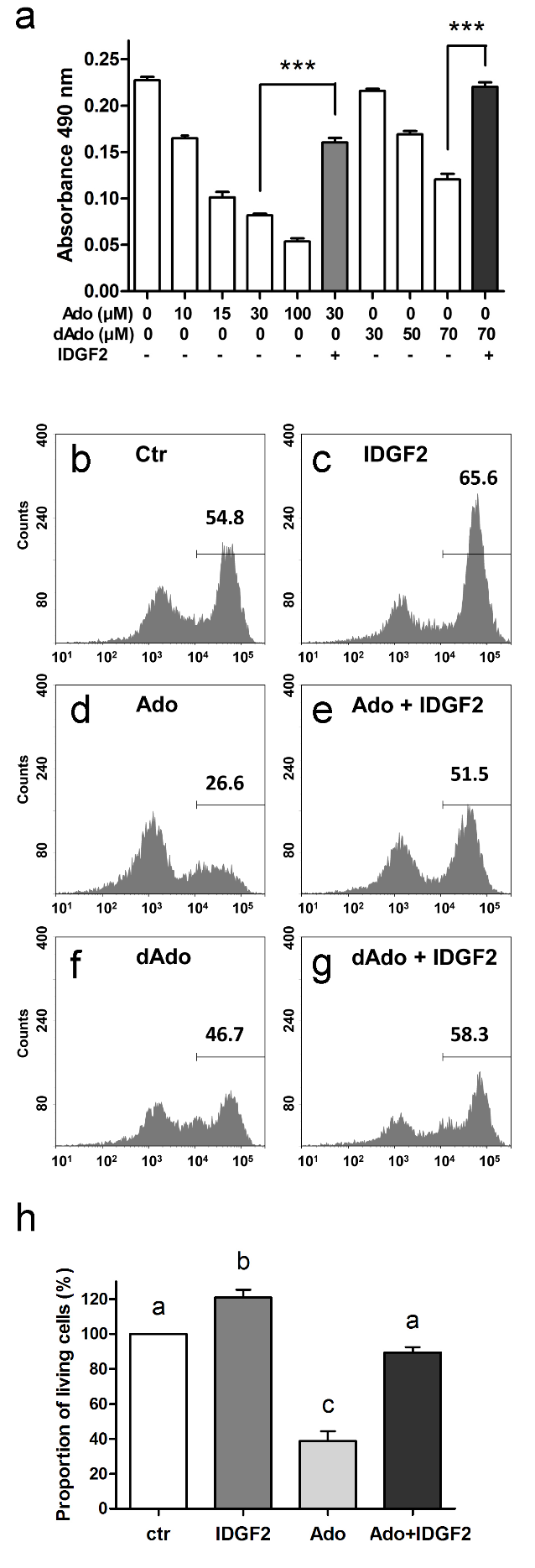
***

**Figure S5**. **Cytotoxic effect of Ado and dAdo can be antagonized by IDGF2.**  (**a**) MTS cell viability assay performed on cells treated with 0, 10, 15, 30 or 100 μM Ado or with 0, 30, 50 or 70 μM dAdo show a concentration-dependent decrease in MTS staining as well as the rescue of 30 μM Ado (grey bar) or 70 μM dAdo (black bar) treated Cl.8+ cells with IDGF2 (16 µg/ml). Data in the graphs are presented as mean ± SEM (n = 5). Significant differences (*** p < 0.001) between highlighted treatments are indicated by asterisk and was evaluated by Student’s t-test. (**b-g**) mitochondrial polarity was assessed by flow cytometric analysis of TMRE stained cells. Cl.8+ cells in MM (**b** and **c**) or MM plus Ado (30 μM) (**d** and **e**) and dAdo (70 μM) (**f**, **g**) in the absence (**b**, **d**, **f**) or presence (**c**, **e**, **g**) of IDGF2 (16 μg/ml). The total cell population was gated on TMRE positive cells; the numbers represent the proportion of these cells. (**h**) Effects of IDGF2 on the viability of cells treated by Ado. The graph summarizes three cell cytometry experiments. Significant differences were evaluated by ANOVA followed by Tukey test and are indicated by different letters (p < 0.05).

**Figure S6**. **Effect of IDGF2 on Ado uptake and ATP synthesis and deleterious effects of dAdo and resveratrol on ATP synthesis.** (**a**) IDGF2 (5 or 20 μg/ml) does not influence H^3^-Ado (10 μM) uptake by Cl.8+ cells. Dipy (Dipyridamole) a known inhibitor of equilibrative Ado transport serve as positive control (**b**) IDGF2 (16 μg/ml) does not decrease ATP synthesis in Cl.8+ cells treated with Ado (30 μM). (**c**) Decrease of ATP synthesis in Cl.8+ cells treated with dAdo or resveratrol. Data in the graphs are presented as mean ± SEM (n = 3). Significant differences were evaluated by ANOVA followed by Tukey test and are indicated by different letters (p < 0.05).

*
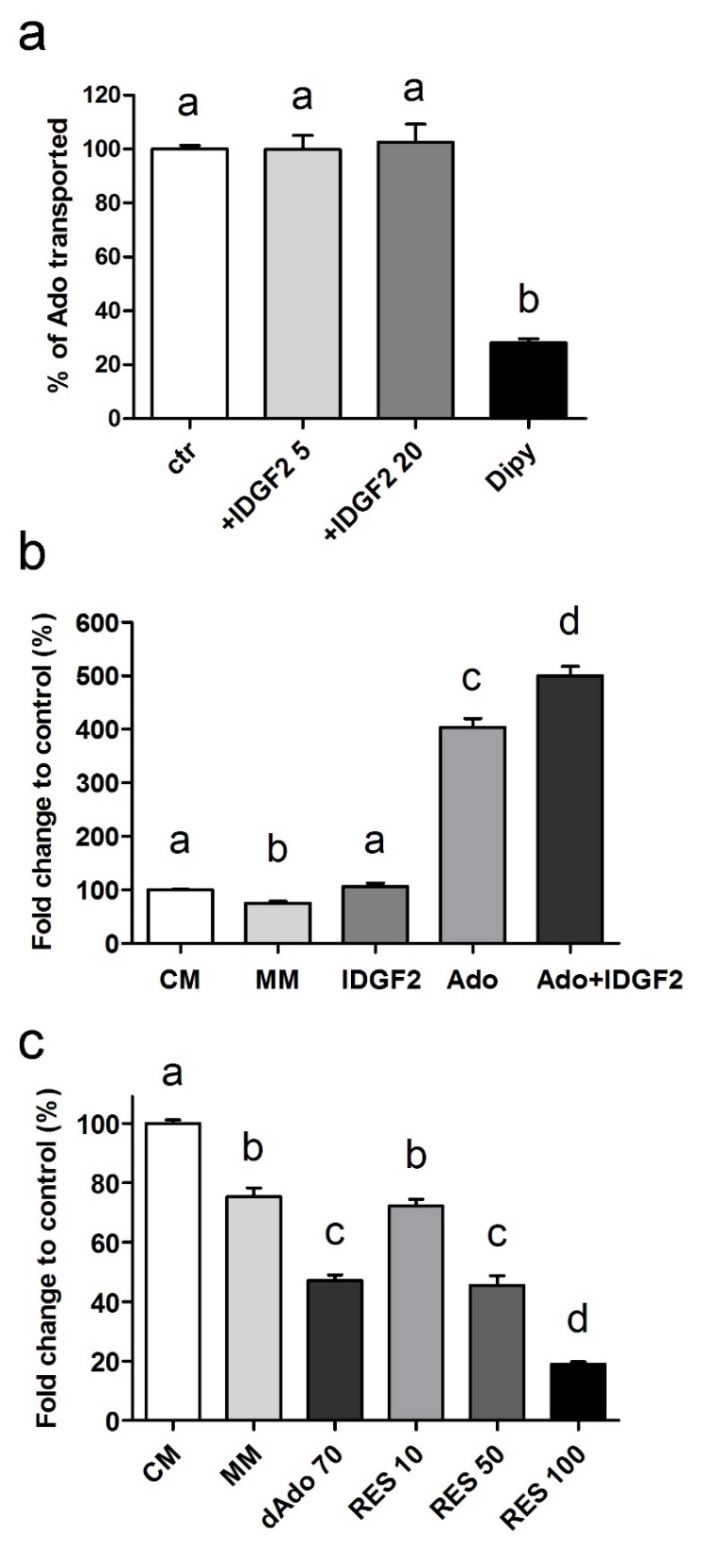
*

**Figure S7**.**The effects of serum (2%) and fly extracts (2.5%) on Cl.8+ cells in MM treated with Ado (100 µM), resveratrol (RES, 100 µM) and rotenone (ROT, 0.01 μM).** Mitochondrial polarity was assessed by flow cytometric analysis of TMRE stained cells. Numbers represent the proportion of viable cells.

*
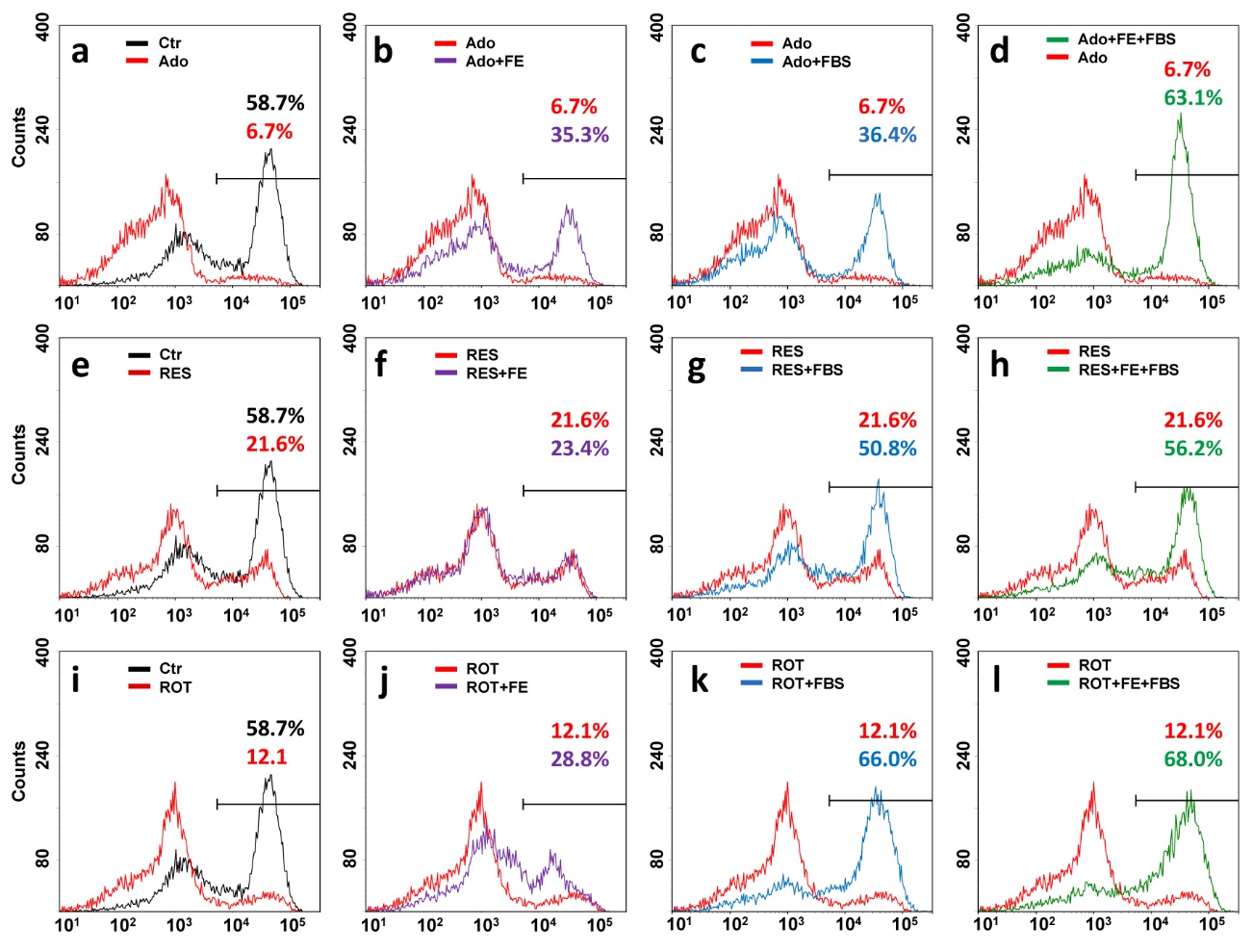
*

**Figure S8.** **Venn diagram summarizing the results of microarray analysis.** Diagram compares numbers of significantly (p < 0.05, |logFC| > 0.8) regulated genes in Cl.8+ cells treated with IDGF2 (16 µg/ml), Ado (50 µM) or IDGF2 (16 µg/ml) + Ado (50 µM). Lists of genes are shown in Tables S2-4.


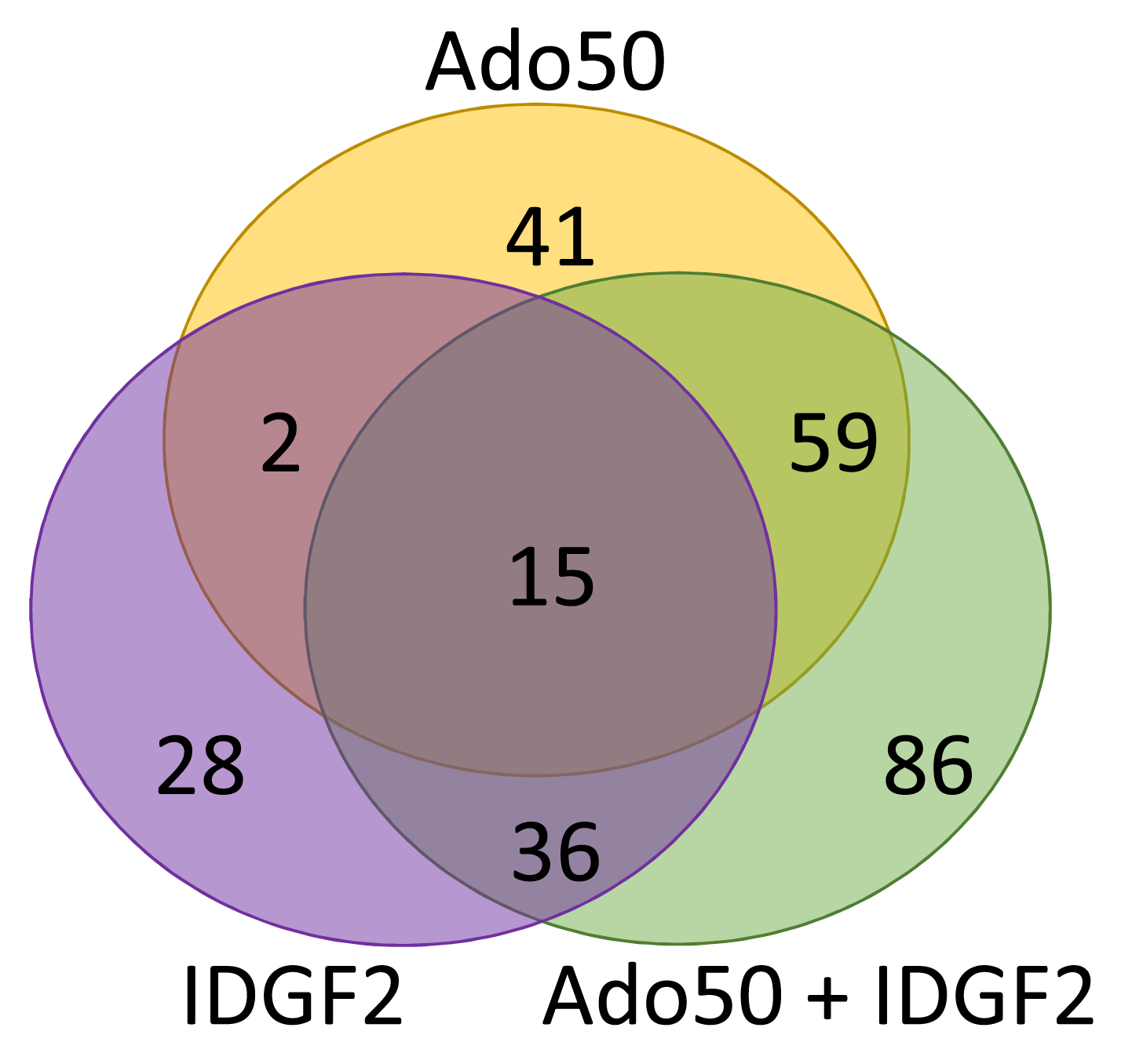


**Figure S9. *Idgf2* transcript expression.** Wild‐type third instar larval tissues showing in situ hybridization with digoxigenin‐labeled antisense RNA probe (**a,** **c**), for *Idgf2*. Strongest expression signal was detected in fat body (**a**), whereas garland cells (red tracing line) attached to proventriculus do not show any signal (**c**). Corresponding negative control with sense RNA probes are shown in (**b, d**). Scale bars: 50 μm.

**
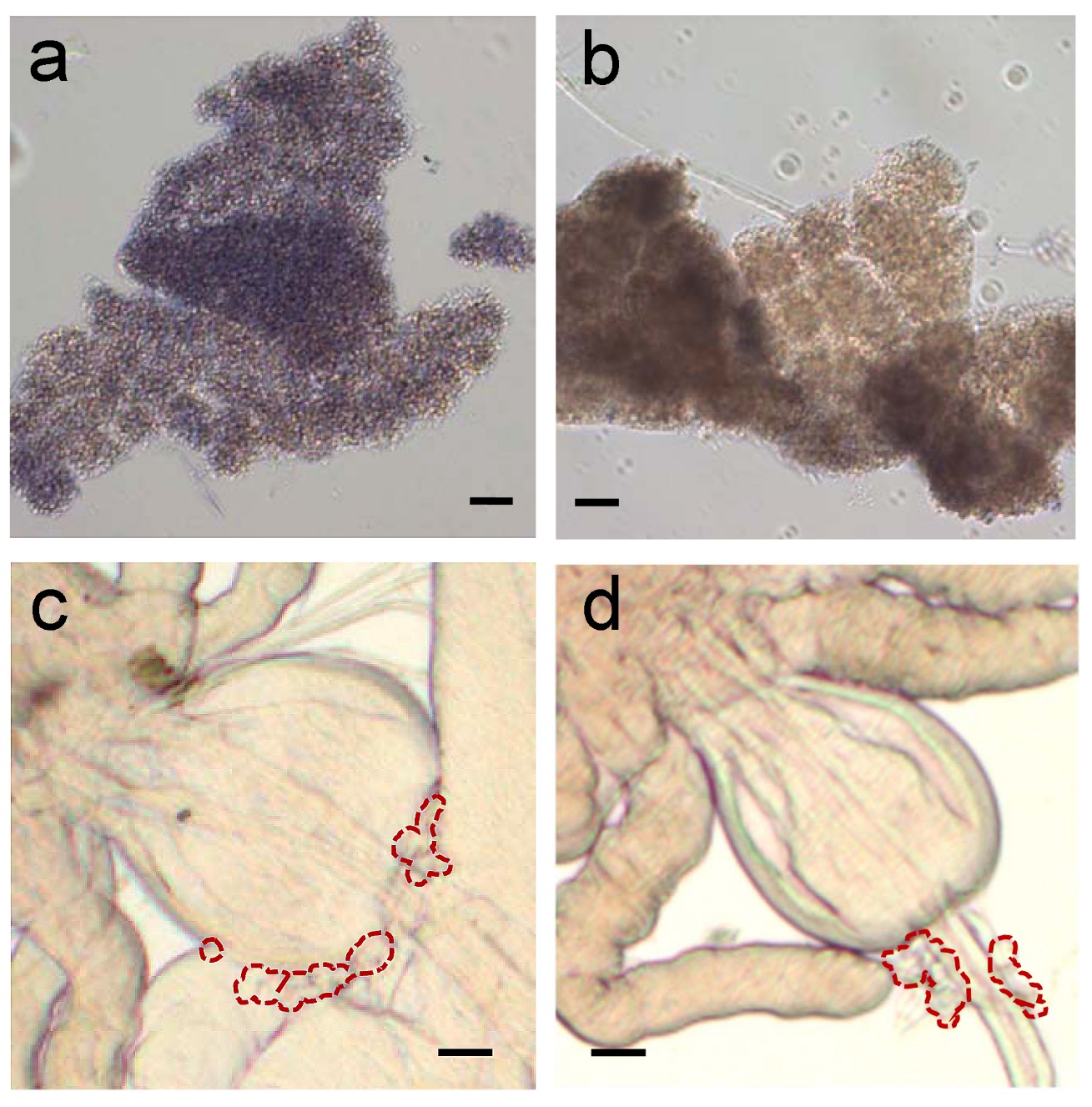
**

**Figure S10. The effects of IDGF2 on S2 cells**. The cells were grown in chemically defined media (**a**, **b**), or treated with Ado 50 μM (**c**, **d**) and Ado 100 μM (**e**, **f**). Mitochondtial membrane potential was assessed by flow cytometric analysis of TMRE stained cells. Numbers represent the proportion of viable cells.

*
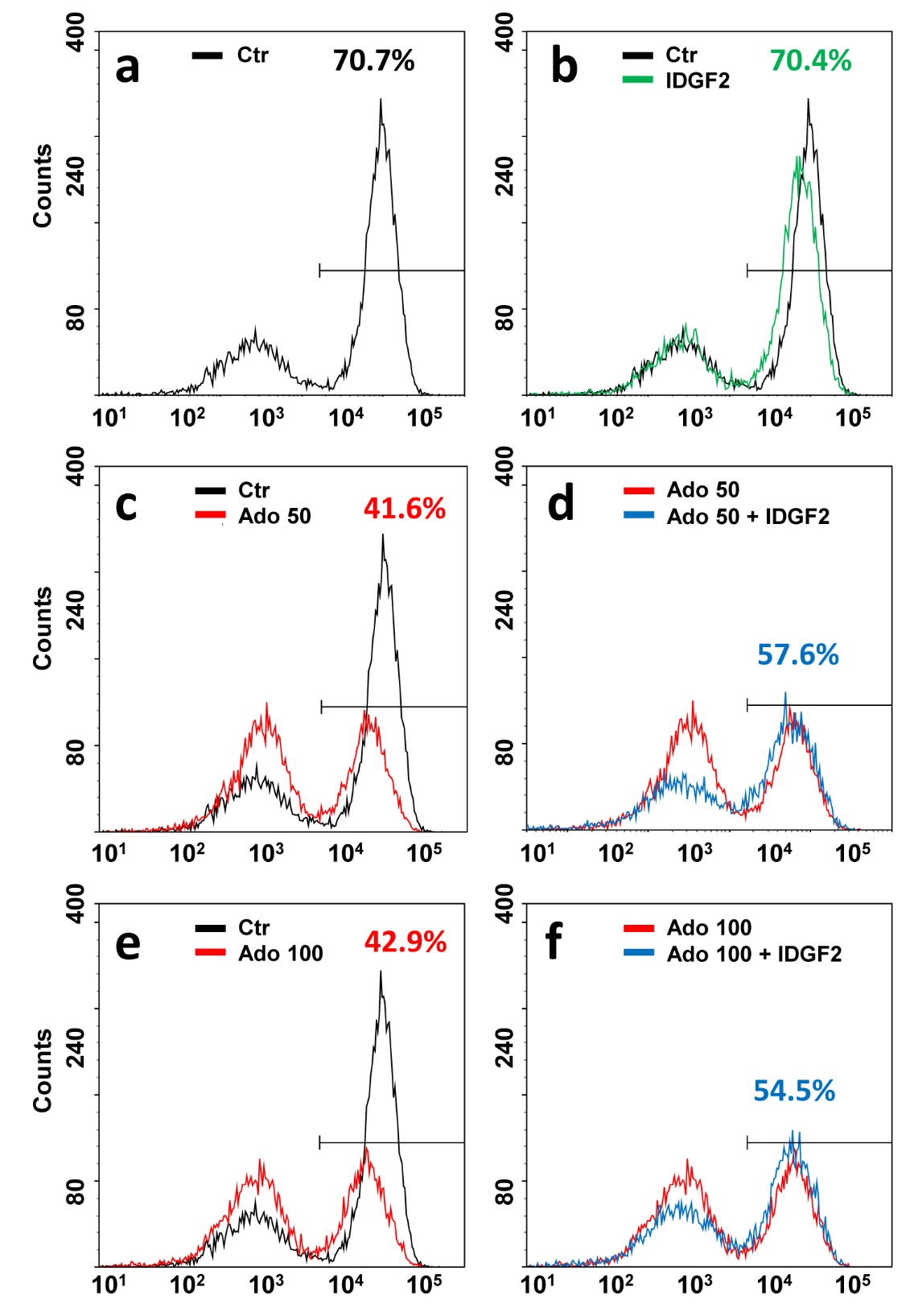
*

**Figure S11. Representative full-length western blot images from Fig. 4g. and northern blot images from Fig. 7b** (**a**) cropped western blot from Fig. 4g.; (**b**, **c**, **d** and **e**) full-length western blots; (**f)** cropped northern blots (Fig7b); **(g, h** and **i)** full length northern blots.


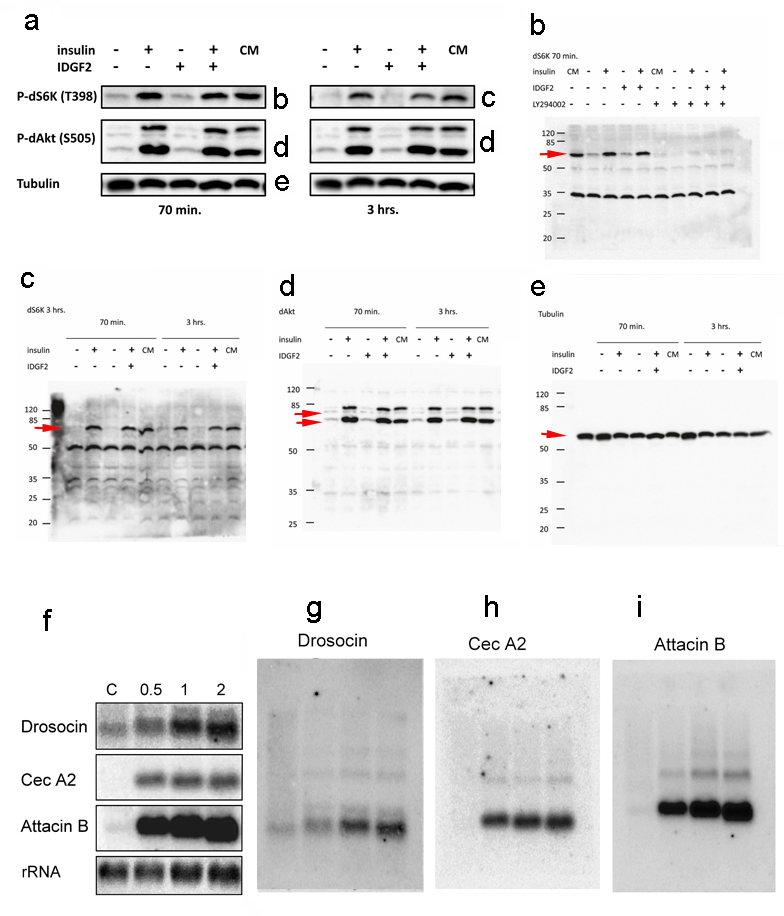


**Table S1.** List of primers used in this study

| **Gene** | **Primer name** | **Sequence** | **Reference** |
| --- | --- | --- | --- |
|  | | | |
| **Idgf2** | forward Idgf2::GFP recombineering | AGTGCTCAGGCGATAAGTATCCCATTCTGCGAGCCATCAAATATCGTCTAGAAGTGCATACCAATCAGGACCCGC | This study |
|  | reverse Idgf2::GFP recombineering | ATAATTGATAATTGTTTATTTAAAGTATTAAATGTTATGTTTCGTTTTACTTGTCGTCGTCATCCTTGTAGTCA | This study |
|  | Idgf2EcoF | TGAATTCATCAtgaaggcgtggatctggtt | This study |
|  | Idgf2R1 | CTTCTGAGATGAGTTTTTGTTCTAGACGATATTTGATGGCTCG | This study |
|  | MycXba | GTTCTAGATCACAGATCCTCTTCTGAGATGAGTTTTTGTTC | This study |
|  | | | |
| **Ribosomal protein L32** | rp49 fw | CTTCATCCGCCACCAGTC | Kucerova et al., 2015 |
|  | rp49 rev | GGCGACGCACTCTGTTGT | Kucerova et al., 2015 |
|  | | | |
| **Imaginal disc growth factor 2** | Idgf2 fw | GCTACTATGACTCTTCGAGTTACACC | This study |
|  | Idgf2 rev | TCCAGATCGGGATTGAGC | This study |
|  | | | |
| **Attacin-A** | AttA fw | TGGTCATGGTGCCTCTTTG | Arefin et al. 2014 |
|  | AttA rev | GATTGTGTCTGCCATTGTTGA | Arefin et al. 2014 |
|  | | | |
| **Attacin-D** | AttD fw | CGGAGTAAGGGTCGGTGAT | This study |
|  | AttD rev | GCCATGCTGCAGTGAGAGT | This study |
|  | | | |
| **Cecropin A1** | CecA1 fw | CTTCGTTTTCGTCGCTCTC | This study |
|  | CecA1 rev | TTTTCTTGCCAATTTTCTTCAG | This study |
|  | | | |
| **Homeodomain interacting protein kinase** | Hipk fw | AGTCGCCGGCTCATCATA | This study |
|  | Hipk rev | CCTTCACCCGCTTCTTCAC | This study |
|  | | | |
| **Zn finger homeodomain 1** | Zfh1 fw | ATCCCGATCTGCCCTATG | This study |
|  | Zfh1 rev | GCCTTCGGACACTCTATGC | This study |
|  | | | |
| **Relish** | Rel fw | CTATGTGGCGCAATTTATCAAC | This study |
|  | Rel rev | TGGCCTCACGCTCTGTCTC | This study |
|  | | | |
| **Peptidoglycan recognition protein LB** | PGRP-LB fw | GGCGATGGCATGATTTACA | This study |
|  | PGRP-LB rev | GCGGCAGTTCGGTTCTC | This study |

**Table S2**. List of 81 genes differentially expressed upon IDGF2 treatment of Cl.8+ cells. The genes are arranged into classes according to their GO terms. The statistical threshold was set to p<0.05 and the threshold of induction was set to 1.74.

| **SYMBOL** | **GENE NAME** | **logFC** | **GO term** |
| --- | --- | --- | --- |
| **immune response** | | | |
|
| AttA | Attacin-A | 2.91 / 6.54 | antibacterial humoral response |
| AttC | Attacin-C | 3.44 | antibacterial humoral response |
| AttD | Attacin-D | 5.22 | antibacterial humoral response |
| CecA1 | Cecropin A1 | 6.22 | antibacterial humoral response |
| CecA2 | Cecropin A2 | 5.08 | antibacterial humoral response |
| CecB | Cecropin B | 2.09 | antibacterial humoral response |
| CecC | Cecropin C | 5.18 / 5.79 | antibacterial humoral response |
| Dpt | Diptericin | 1.18 | antibacterial humoral response |
| DptB | Diptericin B | 4.91 | antibacterial humoral response |
| Dro | Drosocin | 4.15 | antibacterial humoral response |
| FBgn0030309 | CG1572 | 1.14 | hemocyte proliferation |
| Mtk | Metchnikowin | 2.08 | humoral immune response |
| PGRP-LB | Peptidoglycan recognition protein LB | 4.17 | response to bacterium |
| PGRP-SA | Peptidoglycan recognition protein SA | 1.43 | response to bacterium |
| PGRP-SD | Peptidoglycan recognition protein SD | 3.21 | response to bacterium |
| pirk | poor Imd response upon knock-in | 2.1 | negative regulation |
| Rel | Relish | 1.25 | innate immune response |
| vir-1 | virus-induced RNA 1 | 0.83 | response to virus |
| **metabolic process** | | | |
|
| FBgn0033388 | CG8046 | 1.06 | metabolic process |
| FBgn0037344 | CG2926 | 0.8 | regulation of gene expression |
| FBgn0037482 | CG10055 | 0.9 | primary metabolic process |
| Gclc | Glutamate-cysteine ligase | 0.99 | biosynthetic process; |
| gish | gilgamesh | 1.2 | protein phosphorylation |
| GstD2 | Glutathione S transferase D2 | 0.99 | metabolic and cellular process |
| Psa | Puromycin sensitive aminopeptidase | 0.84 | regulation of proteolysis |
| qin | qin | 0.85 | regulation of gene expression |
| scaf | scarface | 1.18 | protein metabolic process |
| Spn4 | Serine protease inhibitor 4 | 1.12 | regulation of proteolysis |
| Sulf1 | Sulfated | 1.02 | metabolic and cellular process |
| y | yellow | 0.86 | biosynthetic process |
|  | | | |
| **localization - transport** | | | |
|
|
| AnnIX | Annexin IX | 1.06 | endosomal transport |
| CASK | CASK ortholog | 0.97 | protein localization |
| CG4928 | UNC93-like protein | 1.34 | transport |
| FBgn0038414 | CG6901 | 1.04 | transmembrane transport |
| FBgn0039644 | CG11897 | 0.84 | transport |
| Mec2 | CG7635 | 1.06 | nephrocyte filtration |
| Mvl | Malvolio | 1.56 | metal ion transport |
| Oatp74D | Organic anion transporting polypeptide 74D | 0.97 | transport |
| Pmp70 | Peroxisomal Membrane Protein 70 kDa | 0.9 | transmembrane transport |
| RabX1 | RabX1 | 1.35 | vesicle-mediated transport |
| spri | sprint | 1.23 | vesicle-mediated transport |
| Trn | Transportin | 0.96 | intracellular protein transport |
| **morphogenesis and development** | | | |
|
| CAP | CG18408 | 0.95 | system development |
| CrebA | Cyclic-AMP response element binding protein A | 1.4 | system development |
| FBgn0033889 | CG6701 | 1.09 | nervous system development |
| FBgn0260965 | CG42588 | 0.97 | nervous system development |
| FBgn0263346 | CG43427 | 0.92 | wing disc development |
| FBgn0263993 | CG43736 | 0.88 | morphogenesis and development |
| fng | fringe | -0.8 | imaginal disc morphogenesis |
| foi | fear-of-intimacy | 2.09 | system development |
| fra | frazzled | 0.84 | nervous system development |
| glec | gliolectin | 1.19 | nervous system development |
| hipk | homeodomain interacting protein kinase | 1.51 | imaginal disc morphogenesis |
| kug | kugelei | 1.08 | system development |
| Mob2 | CG11711 | 0.97 | system development |
| Nrg | Neuroglian | 0.95 | nervous system development |
| pyd | polychaetoid | 0.85 | morphogenesis and development |
| spen | split ends | 1.48 | imaginal disc morphogenesis |
| Wnk | CG7177 | 0.81 | system development |
| zfh1 | Zn finger homeodomain 1 | 1.12 | garland nephrocyte differentiation |
|  | | | |
|  | | | |
|  | | | |
| **cellular component organization or biogenesis** | | | |
|
|
| Jupiter | CG31363 | 0.99 | cytoskeleton organization |
| Map205 | Microtubule-associated protein 205 | 0.86 | cytoskeleton organization |
| mask | multiple ankyrin repeats single KH domain | 1.82 | cytoskeleton organization |
| Mmp1 | Matrix metalloproteinase 1 | 2.51 | membrane organization |
| nuf | nuclear fallout | 0.92 | cytoskeleton organization |
| Rac2 | CG8556 | 0.97 | cytoskeleton organization |
| RhoGAP18B | Rho GTPase activating protein at 18B | 0.87 | cytoskeleton organization |
| spir | spire | 1.47 | cytoskeleton organization |
| trio | CG18214 | 0.81 | cytoskeleton organization |
| **response to stimulus** | | | |
|
| Ect4 | Ectoderm-expressed 4 | 0.87 | signal transduction |
| FBgn0046763 | CG17278 | 1.05 | signal transduction |
| FBgn0263706 | CG43658 | 1.34 | signal transduction |
| FBgn0264339 | CG43795 | 0.81 | signal transduction |
| for | foraging | 1.21 | response to food |
| Gprk2 | G protein-coupled receptor kinase 2 | 1.09 | signal transduction |
| Rtnl1 | Reticulon-like1 | 0.94 | response to chemicals |
| sda | slamdance | 1.82 | mechanical stimulus |
| **unknown** | | | |
|
| FBgn0036419 | CG13482 | 2.36 | unknown |
| FBgn0052436 | CG32436 | -0.89 | unknown |
| FBgn0266101 | CG44838 | 1.24 | unknown |
| SP1173 | CG10121 | 0.82 | unknown |

**Table S3.** List of 117 genes differentially expressed upon 50 μM Ado treatment of Cl.8+ cells. The genes are arranged into classes according to their GO terms. The statistical threshold was set to p<0.05 and the threshold of induction was set to 1.74.

| **SYMBOL** | **GENE NAME** | **logFC** | **GO term** |
| --- | --- | --- | --- |
| **immune response** | | | |
|
| FBgn0033459 | CG12744 | 0.87 | response to fungus |
| pirk | poor Imd response upon knock-in | 0.8 | negative regulation |
| vir-1 | virus-induced RNA 1 | 1.26 | response to virus |
| **metabolic process** | | | |
|
| bip2 | CG2009 | 1.01 | biosynthetic process |
| bun | bunched | 1.89 | primary metabolic process |
| crp | cropped | 1.74 | biosynthetic process |
| ctp | cut up | 1.09 | metabolic or cellular process |
| dm | diminutive | 1.71 | biosynthetic process |
| DOR | Diabetes and obesity regulated | 0.99 | biosynthetic process |
| exba | extra bases | 0.82 | protein dephosphorylation |
| FBgn0015351 | CG14906 | 0.91 | biosynthetic process |
| FBgn0028540 | CG9008 | 0.91 | metabolic and cellular process |
| FBgn0030316 | CG11695 | -0.82 | biosynthetic process |
| FBgn0033204 | CG2065 | 0.85 | protein phosphorylation |
| FBgn0036403 | CG6661 | 1.06 | oxidation-reduction process |
| FBgn0036556 | CG5830 | 1.18 | protein acetylation |
| FBgn0037973 | CG18547 | 0.8 | oxidation-reduction process |
| FBgn0038595 | CG7142 | 1.17 | proteolysis |
| FBgn0038730 | CG6300 | 1.43 | primary metabolic process |
| FBgn0051633 | CG31633 | 1.57 | proteolysis |
| gem | gemini | 0.81 | biosynthetic process |
| gish | gilgamesh | 1.36 | protein phosphorylation |
| GLS | Glutaminase | 0.99 | biosynthetic process |
| GstE1 | Glutathione S transferase E1 | 1.09 | metabolic and cellular process |
| GstO3 | Glutathione S transferase O3 | 0.82 | metabolic and cellular process |
| Hsp26 | Heat shock protein 26 | 1.07 | protein folding |
| Hsp27 | Heat shock protein 27 | 0.94 | protein folding |
| Hsp67Bc | Heat shock gene 67Bc | 1.15 | protein folding |
| Hsp68 | Heat shock protein 68 | 1.06 | protein folding |
| Hsp70Ba | Heat-shock-protein-70Ba | 1.6 | protein folding |
| chm | chameau | 1.38 | protein acetylation |
| lolal | lola like | 0.9 | regulation of gene expression |
| MED10 | Mediator complex subunit 10 | 0.82 | biosynthetic process |
| MED26 | Mediator complex subunit 26 | 1.04 | biosynthetic process |
| Mocs1 | Molybdenum cofactor synthesis 1 ortholog | 0.86 | biosynthetic process |
| Paip2 | polyA-binding protein interacting protein 2 | 1.11 | biosynthetic process |
| Pka-C3 | cAMP-dependent protein kinase 3 | 1.66 | primary metabolic process |
| Ppat-Dpck | Bifunctional Phosphopantetheine adenylyltransferase - Dephospho-CoA kinase | -1.03 | regulation of growth |
| PRL-1 | PRL-1 phosphatase | 1.23 | metabolic process |
| rho | rhomboid | 0.97 | protein metabolic process |
| Spn4 | Serine protease inhibitor 4 | 0.86 | regulation of proteolysis |
| tws | twins | 0.87 | regulation of proteolysis |
| **localization - transport** | | | |
|
| Best1 | Bestrophin 1 | 0.87 | transport |
| corn | cornetto | 1.41 | vesicle-mediated transport |
| FBgn0031645 | CG3036 | 1.68 | transport |
| FBgn0039644 | CG11897 | 0.93 | transport |
| FBgn0051272 | CG31272 | 0.89 | transport |
| glob1 | globin 1 | 1.57 | vesicle-mediated transport |
| Nup153 | Nucleoporin 153 | 1.06 | transport |
| Rala | Ras-related protein | 1.42 | transport |
| rin | rasputin | 1.08 | transport |
| sky | skywalker | 1.21 | vesicle-mediated transport |
| Syx1A | Syntaxin 1A | 1.17 | vesicle-mediated transport |
| Vap-33-1 | VAMP-associated protein 33kDa | 1 | transport |
| yin | CG44402 | -0.88 | vesicle-mediated transport |
| ZnT77C | Zinc transporter 77C | 1.16 | transport |
| **morphogenesis and development** | | | |
|
| a | arc | 1 | morphogenesis and development |
| Akap200 | A kinase anchor protein 200 | 0.9 | system development |
| akirin | CG8580 | 1.49 | morphogenesis and development |
| CAP | CG18408 | 1.05 | system development |
| comm2 | CG7554 | 0.88 | nervous system development |
| Cul-2 | Cullin-2 | 0.87 | morphogenesis and development |
| dlp | dally-like | 1.45 | cell development |
| fax | failed axon connections | 1.51 | nervous system development |
| FBgn0036814 | CG14073 | 0.8 | morphogenesis and development |
| FBgn0039316 | CG11893 | 0.87 | morphogenesis and development |
| FBgn0263346 | CG43427 | 0.86 | wing disc development |
| FBgn0263993 | CG43736 | 0.94 | morphogenesis and development |
| glec | gliolectin | 1.33 | nervous system development |
| hig | hikaru genki | 1.28 | system development |
| hipk | homeodomain interacting protein kinase | 1.56 | imaginal disc morphogenesis |
| kay | kayak | 0.95 | morphogenesis and development |
| lama | lamina ancestor | 1.13 | cell development |
| Nrt | Neurotactin | 0.97 | nervous system development |
| pnt | pointed | 0.96 | cell differentiation |
| S | Star | 1.11 | system development |
| Socs36E | Suppressor of cytokine signaling at 36E | 1.07 | embryonic morphogenesis |
| sprt | sprite | 1.35 | garland nephrocyte differentiation |
| stau | staufen | 0.85 | oocyte development |
| Swip-1 | Swiprosin-1 | 1.11 | morphogenesis and development |
| Ten-m | Tenascin major | 1.15 | morphogenesis and development |
| uzip | unzipped | 0.95 | nervous system development |
| **cellular component organization or biogenesis** | | | |
|
| Fhos | Formin homology 2 domain containing | 1.17 | cellular component movement |
| mask | multiple ankyrin repeats single KH domain | 1.59 | cytoskeleton organization |
| Mmp1 | Matrix metalloproteinase 1 | 1.33 | membrane organization |
| nuf | nuclear fallout | 1 | cytoskeleton organization |
| pyd | polychaetoid | 0.95 | cytoskeleton organization |
| Rcd2 | Reduction in Cnn dots 2 | 1.79 | cell cycle process |
| spir | spire | 1.07 | cytoskeleton organization |
| **response to stimulus** | | | |
|
| Btk29A | Btk family kinase at 29A | 0.82 | signal transduction |
| dsd | distracted | 0.87 | signal transduction |
| FBgn0039419 | CG12290 | 0.92 | signal transduction |
| FBgn0046763 | CG17278 | 0.89 | signal transduction |
| FBgn0051694 | CG31694 | 1.14 / 1.19 | signal transduction |
| itp | ion transport peptide | 0.95 | defense response |
| pes | peste | 1.16 | defense response |
| Piezo | CG44122 | 0.93 | signal transduction |
| Traf4 | TNF-receptor-associated factor 4 | 0.87 | signal transduction |

| **unknown** | | | |
| --- | --- | --- | --- |
|
| bip1 | CG7574 | 0.9 | unknown |
| FBgn0031474 | CG2991 | 1.06 | unknown |
| FBgn0032197 | CG5694 | 1.22 | unknown |
| FBgn0032400 | CG6770 | 2.18 | unknown |
| FBgn0032805 | CG10337 | 0.8 | unknown |
| FBgn0033458 | CG18446 | 0.8 | unknown |
| FBgn0033945 | CG12868 | 1.21 | unknown |
| FBgn0035996 | CG3448 | 1.32 | unknown |
| FBgn0036419 | CG13482 | 0.91 | unknown |
| FBgn0038682 | CG5835 | 0.84 | unknown |
| FBgn0040837 | CG8620 | 1.17 | unknown |
| FBgn0050460 | CG30460 | 0.9 | unknown |
| FBgn0250869 | CG42240 | 2.04 | unknown |
| FBgn0259711 | CG42365 | 0.91 | unknown |
| FBgn0266101 | CG44838 | 1.47 | unknown |
| fok | fledgling of Klp38B | 1.35 | unknown |
| l(1)G0469 | lethal (1) G0469 | 1.1 | unknown |
| Uhg1 | U snoRNA host gene 1 | 1.57 | unknown |
| Xrp1 | CG17836 | 0.95 | unknown |

**Table S4.** List of 196 genes differentially expressed upon simultaneous treatment of IDGF2 plus 50 μM Ado in Cl.8+ cells. The genes are arranged into classes according to their GO terms. The statistical threshold was set to p<0.05 and the threshold of induction was set to 1.74. Genes exclusively expressed upon simultaneous treatment of IDGF2 plus 50 μM Ado are shown in gray.

| **SYMBOL** | **GENENAME** | **logFC** | **GO term** |
| --- | --- | --- | --- |
| **immune response** | | | |
|
| AttA | Attacin-A | 2.3 / 6.0 | antibacterial humoral response |
| AttC | Attacin-C | 3.86 | antibacterial humoral response |
| AttD | Attacin-D | 5.33 | antibacterial humoral response |
| cact | cactus | 1.06 | immune response |
| CecA1 | Cecropin A1 | 6.52 | antibacterial humoral response |
| CecA2 | Cecropin A2 | 5.98 | antibacterial humoral response |
| CecB | Cecropin B | 2.32 | antibacterial humoral response |
| CecC | Cecropin C | 5.1 / 5.7 | antibacterial humoral response |
| Dpt | Diptericin | 1.34 | antibacterial humoral response |
| DptB | Diptericin B | 4.82 | antibacterial humoral response |
| Dro | Drosocin | 3.66 | antibacterial humoral response |
| FBgn0030309 | CG1572 | 0.9 | hemocyte proliferation |
| FBgn0033459 | CG12744 | 0.99 | response to fungus |
| FBgn0259735 | CG42389 | 0.81 | immune response |
| LysX | Lysozyme X | 0.89 | humoral immune response |
| Mtk | Metchnikowin | 1.77 | humoral immune response |
| PGRP-LB | Peptidoglycan recognition protein LB | 3.13 | response to bacterium |
| PGRP-SA | Peptidoglycan recognition protein SA | 0.97 | response to bacterium |
| PGRP-SD | Peptidoglycan recognition protein SD | 2.88 | response to bacterium |
| pirk | poor Imd response upon knock-in | 2.63 | negative regulation |
| Rel | Relish | 1.09 | innate immune response |
| TepIV | Thiolester containing protein IV | 1.2 | antibacterial humoral response |
| vir-1 | virus-induced RNA 1 | 1.94 | response to virus |
| **metabolic process** | | | |
|
| alph | alphabet | 1.04 | primary metabolic process |
| bun | bunched | 2.02 | primary metabolic process |
| CalpB | Calpain-B | 0.9 / 1.1 | proteolysis |
| Clamp | Chromatin-linked adaptor for MSL proteins | 0.84 | regulation of gene expression |
| crp | cropped | 1.83 | biosynthetic process |
| Cyp6w1 | CG8345 | -1.21 | oxidation-reduction process |
| dm | diminutive | 1.74 | biosynthetic process |
| DOR | Diabetes and obesity regulated | 0.91 | biosynthetic process |
| EloA | Elongin A | 0.8 | regulation of gene expression |
| Ets21C | Ets at 21C | 0.97 | regulation of gene expression |
| FBgn0015351 | CG14906 | 1.38 | biosynthetic process |
| FBgn0029856 | CG11700 | 1.07 | protein metabolic process |
| FBgn0030332 | CG9360 | 0.97 | biosynthetic process |
| FBgn0033204 | CG2065 | 1.36 | protein phosphorylation |
| FBgn0034583 | CG10527 | 0.87 | methylation |
| FBgn0036403 | CG6661 | 1.68 | oxidation-reduction process |
| FBgn0036828 | CG6841 | 0.9 | regulation of gene expression |
| FBgn0036837 | CG18135 | -0.95 | lipid metabolic process |
| FBgn0037973 | CG18547 | 0.82 | oxidation-reduction process |
| FBgn0038381 | CG3303 | 0.9 | proteolysis |
| FBgn0038470 | CG18213 | 0.87 | regulation of gene expression |
| FBgn0038595 | CG7142 | 1.08 | proteolysis |
| FBgn0038730 | CG6300 | 0.84 | primary metabolic process |
| FBgn0051633 | CG31633 | 1.34 | proteolysis |
| FBgn0052369 | CG32369 | 0.81 | proteolysis |
| FBgn0052549 | CG32549 | 0.88 | primary metabolic process |
| Fur1 | Furin 1 | 1.03 | proteolysis |
| gem | gemini | 0.85 | biosynthetic process |
| gish | gilgamesh | 1.34 | protein phosphorylation |
| GLS | Glutaminase | 1.21 | biosynthetic process |
| GstD2 | Glutathione S transferase D2 | 1.16 | metabolic and cellular process |
| GstD3 | Glutathione S transferase D3 | 0.92 | metabolic and cellular process |
| GstD4 | Glutathione S transferase D4 | 1.51 | metabolic and cellular process |
| GstE1 | Glutathione S transferase E1 | 1.55 | metabolic and cellular process |
| GstO3 | Glutathione S transferase O3 | 1.27 | metabolic and cellular process |
| Hsc70Cb | CG6603 | 0.98 | protein folding |
| Hsp26 | Heat shock protein 26 | 1.42 | protein folding |
| Hsp27 | Heat shock protein 27 | 1.32 | protein folding |
| Hsp67Bc | Heat shock gene 67Bc | 1.55 | protein folding |
| Hsp68 | Heat shock protein 68 | 1.86 | protein folding |
| Hsp70Bbb | Heat-shock-protein-70Bb | 2.09 | protein folding |
| chm | chameau | 1.68 | protein acetylation |
| Kr-h1 | Kruppel homolog 1 | 0.83 | regulation of gene expression |
| kuz | kuzbanian | 1.4 | proteolysis |
| Mocs1 | Molybdenum cofactor synthesis 1 ortholog | 1.26 | biosynthetic process |
| mus201 | mutagen-sensitive 201 | 0.85 | metabolic process - DNA repair |
| Naam | Nicotinamide amidase | 1.08 | metabolic process |
| Oda | Ornithine decarboxylase antizyme | 0.92 | biosynthetic process |
| Pde8 | Phosphodiesterase 8 | 0.95 | metabolic process |
| PRL-1 | PRL-1 phosphatase | 1.14 | metabolic process |
| Psa | Puromycin sensitive aminopeptidase | 1.36 | regulation of proteolysis |
| qin | qin | 0.88 | regulation of gene expression |
| rho | rhomboid | 1.17 | protein metabolic process |
| scaf | scarface | 1.08 | protein metabolic process |
| Spn4 | Serine protease inhibitor 4 | 1.84 | regulation of proteolysis |
| Spn6 | Serine protease inhibitor 6 | 1.21 | regulation of proteolysis |
| stv | starvin | 0.81 | protein metabolic process |
| y | yellow | 1.4 | biosynthetic process; |
| δTry | deltaTrypsin | 0.82 | proteolysis |
| **localization - transport** | | | |
|
| AnnIX | Annexin IX | 1.22 | endosomal transport |
| Best1 | Bestrophin 1 | 1.25 | transport |
| corn | cornetto | 1.59 | vesicle-mediated transport |
| FBgn0026875 | CG3638 | 1.46 | transport |
| FBgn0027556 | CG4928 | 1.64 | transport |
| FBgn0029896 | CG3168 | 0.85 | transport |
| FBgn0031645 | CG3036 | 1.5 | transport |
| FBgn0032026 | CG7627 | 0.87 | transport |
| FBgn0036043 | CG8177 | 1.62 | transport |
| FBgn0038414 | CG6901 | 1.14 | transmembrane transport |
| FBgn0039644 | CG11897 | 1.43 | transport |
| FBgn0051272 | CG31272 | 1.39 | transport |
| FBgn0052103 | CG32103 | 0.99 | transport |
| Gie | GTPase indispensable for equal segregation of chromosomes | 1.09 | vesicle-mediated transport |
| glob1 | globin 1 | 1.3 | vesicle-mediated transport |
| Klp61F | Kinesin-like protein at 61F | -0.8 | vesicle-mediated transport |
| Mvl | Malvolio | 1.45 | metal ion transport |
| nrv1 | nervana 1 | 0.89 | transport |
| Prestin | CG5485 | 0.96 | transport |
| Rab1 | Rab-protein 1 | 0.81 | vesicle-mediated transport |
| RabX1 | RabX1 | 1.02 | vesicle-mediated transport |
| subdued | subdued | 0.86 | transport |
| Tret1-1 | Trehalose transporter 1-1 | 1.07 | transport |
| Tret1-2 | Trehalose transporter 1-2 | 0.87 | transport |
| Trn | Transportin | 0.86 | intracellular protein transport |
| Vha100-2 | Vacuolar H[+] ATPase subunit 100-2 | -0.82 | proton transport |
| Zip99C | CG7816 | 0.97 | transport |
| ZnT77C | Zinc transporter 77C | 1.21 | transport |
| **morphogenesis and development** | | | |
|
| CAP | CG18408 | 0.8 / 1.5 | morphogenesis and development |
| comm2 | CG7554 | 1.32 | nervous system development |
| cv-2 | crossveinless 2 | 1.0 | nervous system development |
| dally | division abnormally delayed | 0.94 | nervous system development |
| dlp | dally-like | 1.46 | cell development |
| fax | failed axon connections | 1.72 | nervous system development |
| FBgn0051534 | CG43427 | 1.02 | wing disc development |
| foi | fear-of-intimacy | 1.79 | system development |
| glec | gliolectin | 1.58 | nervous system development |
| hig | hikaru genki | 1.42 | system development |
| hipk | homeodomain interacting protein kinase | 1.48 | imaginal disc morphogenesis |
| lama | lamina ancestor | 1.65 | cell development |
| Nrt | Neurotactin | 1.7 | nervous system development |
| Psc | Posterior sex combs | 0.8 | system development |
| S | Star | 1.34 | system development |
| Socs36E | Suppressor of cytokine signaling at 36E | 1.84 | embryonic morphogenesis |
| sprt | sprite | 1.41 | garland nephrocyte differentiation |
| Swip-1 | Swiprosin-1 | 0.97 | morphogenesis and development |
| trbl | tribbles | 0.82 | imaginal disc morphogenesis |
| Tsp42Ef | Tetraspanin 42Ef | 1.03 | nervous system development |
| zfh1 | Zn finger homeodomain 1 | 0.81 | garland nephrocyte differentiation |
| **cellular component organization or biogenesis** | | | |
|
| caps | capricious | 0.95 | cell adhesion |
| Dg | Dystroglycan | 0.84 | cytoskeleton organization |
| FBgn0034160 | CG5550 | 1.36 | cytoskeleton organization |
| Fhos | Formin homology 2 domain containing | 1.04 | cellular component movement |
| Gli | Gliotactin | 1.03 | cytoskeleton organization |
| Incenp | Inner centromere protein | -0.86 | mitotic cell cycle process |
| Kmn1 | kinetochore Mis12-Ndc80 network component 1 | -0.82 | cell cycle process |
| LamC | Lamin C | 0.89 | cytoskeleton organization |
| Lasp | CG3849 | 0.97 | cytoskeleton organization |
| Mcm7 | Minichromosome maintenance 7 | -0.82 | cell cycle process |
| Mmp1 | Matrix metalloproteinase 1 | 3.28 | membrane organization |
| nuf | nuclear fallout | 1.12 | cytoskeleton organization |
| pav | pavarotti | -0.83 | cell cycle process |
| polo | polo | -0.86 | cell cycle process |
| Rcd2 | Reduction in Cnn dots 2 | 2.21 | cell cycle process |
| rhea | CG6831 | 0.92 | cytoskeleton organization |
| rols | rolling pebbles | 0.84 | cytoskeleton organization |
| spir | spire | 1.2 / 1.7 | cytoskeleton organization |
| trio | CG18214 | 0.86 | cytoskeleton organization |
| **response to stimulus** | | | |
|
| 14-3-3zeta | 14-3-3zeta | 0.81 | signal transduction |
| Akt1 | CG4006 | 0.82 | signal transduction |
| Ars2 | CG7843 | 0.91 | response to chemicals |
| Cam | Calmodulin | 1.67 | signal transduction |
| drk | downstream of receptor kinase | 0.81 | signal transduction |
| Ect4 | Ectoderm-expressed 4 | 0.9 / 1.0 | signal transduction |
| FBgn0039419 | CG12290 | 1.01 | signal transduction |
| FBgn0046763 | CG17278 | 0.98 | signal transduction |
| FBgn0051694 | CG31694 | 1.3 / 1.4 | signal transduction |
| for | foraging | 1.46 | response to food |
| Galpha73B | G protein alpha 73B | 1.0 | signal transduction |
| G-oalpha47A | G protein oalpha 47A | 1.34 | signal transduction |
| MESR3 | Misexpression suppressor of ras 3 | -1.35 | signal transduction |
| pes | peste | 1.43 | defense response |
| Pvf3 | PDGF- and VEGF-related factor 3 | 1.12 | signal transduction |
| Rtnl1 | CG33113 | 0.8 / 1.2 | response to stimulus |
| sda | slamdance | 1.85 | mechanical stimulus |
| **unknown** | | | |
|
| Arc2 | CG13941 | 0.82 | unknown |
| bip1 | CG7574 | 1.19 | unknown |
| FBgn0029766 | CG15784 | 0.89 | unknown |
| FBgn0031474 | CG2991 | 1.03 | unknown |
| FBgn0032022 | CG14275 | 0.86 | unknown |
| FBgn0032400 | CG6770 | 2.1 | unknown |
| FBgn0032587 | CG5953 | 0.8 | unknown |
| FBgn0032805 | CG10337 | 1.35 | unknown |
| FBgn0033458 | CG18446 | 1.18 | unknown |
| FBgn0033945 | CG12868 | 0.97 | unknown |
| FBgn0035237 | CG13917 | 0.87 | unknown |
| FBgn0035996 | CG3448 | 1 | unknown |
| FBgn0036419 | CG13482 | 2.74 | unknown |
| FBgn0037016 | CG13252 | 0.89 | unknown |
| FBgn0037746 | CG8478 | -1.08 | unknown |
| FBgn0038638 | CG7702 | 1.87 | unknown |
| FBgn0038682 | CG5835 | 1.83 | unknown |
| FBgn0040837 | CG8620 | 1.5 | unknown |
| FBgn0052541 | CG43759 | 0.87 | unknown |
| FBgn0250869 | CG42240 | 2.03 | unknown |
| FBgn0265185 | CG44250 | 1.46 | unknown |
| FBgn0266101 | CG44838 | 1.21 | unknown |
| fok | fledgling of Klp38B | 1.26 | unknown |
| l(1)G0469 | lethal (1) G0469 | 1.4 | unknown |
| NijA | Ninjurin A | 0.85 | unknown |
| Nop17l | Nop17 like | 0.88 | unknown |
| sip2 | septin interacting protein 2 | -1.1 | unknown |
| SP1173 | CG10121 | 1.25 | unknown |
| Xrp1 | CG17836 | 0.83 | unknown |

**Table S5.** The enriched KEGG pathways of differentially expressed genes in Cl.8+ cells treated with Ado, IDGF and Ado + IDGF2 (FDR-value < 0.05 and |logFC| > 0.4). gSIG = number of differentially expressed genes in pathway; gDET = number of genes in pathway verified in our microarrays; FDR = Fisher's exact test, FDR value; Direction = direction of change: “Up” - mostly upregulated genes were detected, “Down“ - mostly downregulated genes were detected.

| **KEGG** | **Name** | **gSIG** | **gDET** | **FDR** | **Direction** |
| --- | --- | --- | --- | --- | --- |
| **IDGF2** | | | | |  |
|  |
| dme04145 | Phagosome | 3 | 29 | 0.0116 | Up |
| **Ado** | | | | |  |
|  |
| dme00770 | Pantothenate and CoA biosynthesis | 2 | 7 | 0.0193 | Down |
| **Ado+IDGF2** | | | | |  |
|  |
| dme00480 | Glutathione metabolism | 7 | 24 | 0.000062 | Up |
| dme00980 | Metabolism of xenobiotics by cytochrome P450 | 7 | 21 | 0.00028 | Up |
| dme00982 | Drug metabolism - cytochrome P450 | 7 | 20 | 0.00028 | Up |
